# Age and cognitive decline in the UK Biobank

**DOI:** 10.1371/journal.pone.0213948

**Published:** 2019-03-18

**Authors:** Marilyn C. Cornelis, Yamin Wang, Thomas Holland, Puja Agarwal, Sandra Weintraub, Martha Clare Morris

**Affiliations:** 1 Department of Preventive Medicine, Northwestern University Feinberg School of Medicine, Chicago, Illinois, United States of America; 2 Rush Institute for Healthy Aging, Rush University, Chicago, Illinois, United States of America; 3 Mesulam Cognitive Neurology and Alzheimer’s Disease Center, Northwestern University Feinberg School of Medicine, Chicago, Illinois, United States of America; 4 Department of Psychiatry and Behavioral Sciences, Northwestern University Feinberg School of Medicine, Chicago, Illinois, United States of America; Nathan S Kline Institute, UNITED STATES

## Abstract

**Objectives:**

Age-related cognitive decline is a well-known phenomenon after age 65 but little is known about earlier changes and prior studies are based on relatively small samples. We investigated the impact of age on cognitive decline in the largest population sample to date including young to old adults.

**Method:**

Between 100,352 and 468,534 participants aged 38–73 years from UK Biobank completed at least one of seven self-administered cognitive functioning tests: prospective memory (PM), pairs matching (Pairs), fluid intelligence (FI), reaction time (RT), symbol digit substitution, trail making A and B. Up to 26,005 participants completed at least one of two follow-up assessments of PM, Pairs, FI and RT. Multivariable regression models examined the association between age (<45[reference], 45–49, 50–54, 55–59, 60–64, 65+) and cognition scores at baseline. Mixed models estimated the impact of age on cognitive decline over follow-up (~5.1 years).

**Results:**

FI was higher between ages 50 and 64 and lower at 65+ compared to <45 at baseline. Performance on all other baseline tests was lower with older age: with increasing age category, difference in test scores ranged from 2.5 to 7.8%(P<0.0001). Compared to <45 at baseline, RT and Pairs performance declined faster across all older age cohorts (3.0 and 1.2% change, respectively, with increasing age category, P<0.0001). Cross-sectional results yielded 8 to 12-fold higher differences in RT and Pairs with age compared to longitudinal results.

**Conclusions:**

Our findings suggest that declines in cognitive abilities <65 are small. The cross-sectional differences in cognition scores for middle to older adult years may be due in part to age cohort effects.

## Introduction

Age is a key risk factor for cognitive performance. Cognitive decline is common in older ages but recently there has been interest in understanding the age at which significant decline in cognitive abilities begins. Such knowledge has implications for the design of behavioral or pharmacological interventions since they are more likely to work if they are applied when, or even years before, individuals first begin to experience decline [1]. Efforts to date are often based on cross-sectional studies which may be confounded by ‘cohort effects’ [2, 3]. Longitudinal studies suggest evidence of cognitive decline in middle age but that age trajectories differ by sex and cognition domain or task [2, 4–7]. The age at peak performance also varies by task [8] and might be relevant to the age range examined for cognitive decline. Longitudinal data that span many decades generally report minimal cognitive decline before the age of 65 (reviewed in [2])[9], but such studies are rare and also subject to limitations including small sample size, selection attrition and retest or practice effects [3]. Hughes et al [7] examined cognitive decline among ~2,500 participants aged 25 to 95 years at recruitment in the Midlife in the Unites States (MIDUS) study, and all cognitive domains measured showed significant but small declines over 9 years, with differences in the timing and extent of change. The largest analysis to-date included a 10 year follow-up of ~7,400 participants aged 45–70 at recruitment of the Whitehall Study [4]. The design of this study allowed for cross-sectional and longitudinal analysis. For the former analysis, performance on several tests were progressively lower with older age categories. In longitudinal analyses, there was some evidence of greater decline at older ages and of a linear trend in decline with increasing age for some of the tests, particularly in men. However, the cross sectional data considerably overestimated cognitive decline among women but not in men.

UK Biobank is a large population cohort of adults aged 37 to 73 who underwent medical, sociodemographic, mental health and cognitive assessment in 2006–2010 and are being followed up at intervals. The large age-distribution and follow-up enables cross-sectional as well as longitudinal analysis of age as done by Singh-Manoux et al [4] but for a much larger sample. In a subsample of the UK Biobank, Hagenaars et al [10] presented simple age trends in standardized mean cognition scores, but the focus of that report was not age-related cognition, and it included only a subset of tests and participants at baseline. At the time the current study was under review, Kievit et al [11] used structural equation modeling to examine age and declines in fluid intelligence in the UK Biobank and reported a weak but significant association of cross-sectional age on the mean fluid intelligence score, such that older individuals scored slightly lower. The current study also leverages this valuable resource to investigate the impact of age on cognitive decline, as determined by several tests, in the largest population sample to date.

## Materials and methods

Supporting Information (S1 File) provide an expanded description of the UK Biobank which is summarized below.

### Participants

In 2006–2010, the UK Biobank recruited over 502,633 participants aged 37–73 years at 22 centers across England, Wales and Scotland [12]. Approximately 9.2 million individuals were invited, and 5.4% provided full informed consent to participate in UK Biobank and completed a 90 minute assessment that included touchscreen questionnaires on sociodemographic factors, lifestyle and medical history, an in-person interview and physical assessment (S1 Table). Data for the current analysis was downloaded in 2017. This study was covered by the generic ethical approval for UK Biobank studies from the National Research Ethics Service Committee North West–Haydock (approval letter dated 17th June 2011, Ref 11/NW/0382), and all study procedures were performed in accordance with the World Medical Association Declaration of Helsinki ethical principles for medical research.

### Measures and procedures

#### Cognitive function testing [13]

At the assessment center, cognitive functioning was assessed using a 15-min self-administered computerized battery which was developed specifically for the UK Biobank study to enable population-scale cognitive testing that could be administered without researcher supervision [12]. In 2014, participants were invited to complete on-line tests at home. A detailed description of all tests are provided on-line [14, 15] and in S1 File. Brief descriptions are provided below.

*Prospective Memory (PM) Test*: This test was added part-way through the baseline assessment period and completed at the assessment centers. Participants were given the following instructions: ‘At the end of the games we will show you four colored symbols and ask you to touch the blue square. However, to test your memory, we want you to actually touch the Orange Circle instead’. PM refers to the ability to carry out future intentions at a specific time or in response to a specific event [16, 17]. Participants were scored as zero or one, depending on whether they completed the task on first attempt or not.

*Pairs Matching (Pairs) Test*: This episodic visual memory test was completed at the assessment centers. Participants were shown 6 pairs of cards for 5 seconds, which were then turned over. Participants were instructed to select, from recall and in the fewest number of attempts, the pairs of cards that had matching symbols. The total number of errors made during this task until the six pairs of identical cards were touched consecutively was recorded. We restricted our analyses to individuals who finished the test and log (+1) transformed the number of errors for the analysis. During the pilot phase of the UK Biobank, a subset of participants completed this test twice in immediate succession and the intraclass correlation was 0.17 [18].

*Fluid Intelligence (FI) Test*: This test, also referred to as the verbal-numerical reasoning test, was added part-way through the baseline assessment period and completed at the assessment centers. Participants were presented with 13 verbal logic/reasoning-type multiple choice questions and had to answer as many as they could within 2 minutes. Incorrect or unattempted questions were scored as zero. The total number of correct answers (max 13) was used for our analysis. The Cronbach alpha coefficient for these items has been reported elsewhere as 0.62 [10].

*Reaction Time (RT) Test*: For this measure of simple processing speed, participants completed a timed test of symbol matching at the assessment centers. Participants were shown one pair of cards out of a set of 12 pairs. If both cards displayed a matching symbol, participants pressed a response button as quickly as possible. The score on this task was the mean response time in milliseconds (ms) across 4 trials which contained matching pairs. Potential outliers (n = 2751) were truncated to 100 (min) or 1000 (max) ms. Cronbach’s alpha for this task has previously been reported as 0.85 [10].

*Symbol Digit Substitution (SDS) Test*: This test for complex processing speed was completed at follow-up (~2014) on home computers and involves matching numbers to a set of symbols. We used the number of correct substitutions for our analyses. Potential outliers (n = 236) were truncated to 1 (min) or 40 (max) correct substitutions.

*Trail Making Test A and B*: These visual attention tests provide information on visual search, scanning, speed of processing, mental flexibility, and executive functions and were completed at follow-up on home computers (~2014). Participants were asked to connect scattered circles containing a sequence of numbers (Trail A) and then to connect circles containing numbers or letters by alternating between them in ascending sequence (Trail B). We used the time taken to complete these tests for our analyses, and these data were log-transformed.

Subsets of participants completing the baseline visit returned to the assessment centers for up to two follow-up assessments. The first follow-up took place 2012–13 and included 20,346 participants, all living within 35 km of the Stockport Biobank coordinating center. The same region is currently being targeted for a second follow-up which began in 2014 and also involves an imaging component. At the time of the current study, data from 11,923 participants was available. At follow-up visits participants were invited to repeat the PM, FI, Pairs and RT tests. Longitudinal analysis for the current study were restricted to individuals completing the baseline tests and at least one follow-up test.

Because some cognitive tasks were added at different stages of baseline assessment or not until 2014 the number of participants varies across tests (S2 Table). Participants were also invited to complete the FI and Pairs tests on home computers but mean performance for these tests were considerably lower than those completed at the assessment center which warranted concern and thus were not included for the current analysis.

#### Other covariates

Information on several covariates functioning as potential confounders were also collected during the UK Biobank assessment as described in detail previously [12, 19]. For the current analysis we considered baseline smoking status, Townsend deprivation index, education, income, alcohol intake, physical activity, ethnicity, and employment status. APOE carriers(ε4+) and non-carriers(ε4-) were defined using genotyped or imputed genotypes for SNPs rs429358 and rs7412 as described in detail elsewhere [20]. Sample outliers based on heterozygosity and missingness were excluded, as were participants with sex discrepancies between self-reported and inferred sex (using X-chromosome heterozygosity). We limited the genetic analysis to unrelated and, in sensitivity analysis, further to individuals who self-report as “British” and who have very similar ancestral backgrounds based on results of principal component analysis.

### Analyses

A total of 496,042 participants completed at least one of the cognitive function tests at baseline (S2 Table provides sample sizes per test). We excluded 22,419 who self-reported neurological disease at baseline or follow-up that could directly affect cognitive function [18]. The remaining 473,623 for the current analysis were of 38 to 73 years of age, which we categorized into ~5 year age groups: <45, 45–49, 50–54, 55–59, 60–64, and 65+.

We first examined the cross sectional associations between age (6 categories) and each of the cognitive tests at baseline using linear or logistic (PM only) regression adjusting for sex. In multivariable regressions we adjusted for sex, baseline smoking (never, past, current), Townsend index (quartiles), education (university/college degree or less), income (4 levels), alcohol intake (quartiles of servings/week), physical activity (quartiles of moderate/vigorous activity minutes/week), ethnicity (white or non-white) and employment status (employed, retired, other). Age was initially modeled categorically to capture potentially complicated (i.e. non-linear) relationships between age and cognitive decline and also to allow comparison of results with previous studies. However, a linear test for trend was also used to assess whether cognitive scores were progressively lower or higher with increasing age. The <45 age group was chosen as the reference since we hypothesized that this group presents with peak cognitive ability which in turn declines with increasing age [8].

Subsets of the cross-sectional sample returned to the centers for at least one of the two follow-up assessments for FI (N = 7,430), Pairs (N = 25,371), RT (N = 25,739) and PM (N = 7,605) tests (see S3 Table for sample sizes per follow-up). The mean (range) follow-up time from the first assessment was 4.5 (2.1–6.1) years to the second assessment and 6.8 (3.8–9.8) years to the third assessment, with shorter intervals for tests introduced part way through recruitment (S4 Table). For FI, Pairs and RT, linear mixed models with random intercept and time (slope) were used to estimate cognitive decline over follow-up [21]. The first model included time between baseline and assessment as the timescale (years) along with age (6 groups), sex, baseline cognition test score and their interactions with time. The time×age interaction term allows the calculation of the yearly rate of decline by age group with reference to the <45 age group. To assess whether the decline increased or decreased with age we entered the categories of age as a continuous variable in the model. Multivariable models included variables (and their interaction with time) used for cross-sectional analysis but further included measures of potential practice effects: the number of follow-up cognitive function tests completed at the center (1 or 2) and whether participants completed an on-line cognitive function test prior to the second follow-up (applicable to FI and Pairs tests only). Similar results were observed when *excluding* the latter. For longitudinal analysis of PM (a binary test score) we employed equivalent crude and multivariable models described for continuous tests but using generalized estimating equations. Associations with P values < 0.007 (0.05/7 cognition tests) were considered statistically significant.

To allow comparison across cognitive tests, which differ in score range, we also express cognitive difference (cross-sectional) or change (longitudinal) in terms of percentage difference/change as a function of the range of each test (yearly rate of change/range of test×100) [4]. For log transformed test scores (Pairs and Trail tests), percentage *change* is the exponentiated beta coefficients (exp(β)-1) ×100). We also performed analysis stratified by sex, education, baseline cognition test performance, and APOE status and tested for interactions by including cross-products of sex-, education-, baseline- and genotype-age in multivariable regression models.

We investigated attrition and practice effects in the longitudinal analysis of FI and PM tests using a similar approach as that described by Schaie [22] and Ronnlund et al [2], leveraging a new sample (N ≤ 14,460) from the target population that did not complete baseline year cognitive tests (i.e. excluded from our primary analysis) but completed the first follow-up (when baseline completers were re-tested)(see S1 File).

## Results

### Cross-sectional analysis

**Fig 1:**
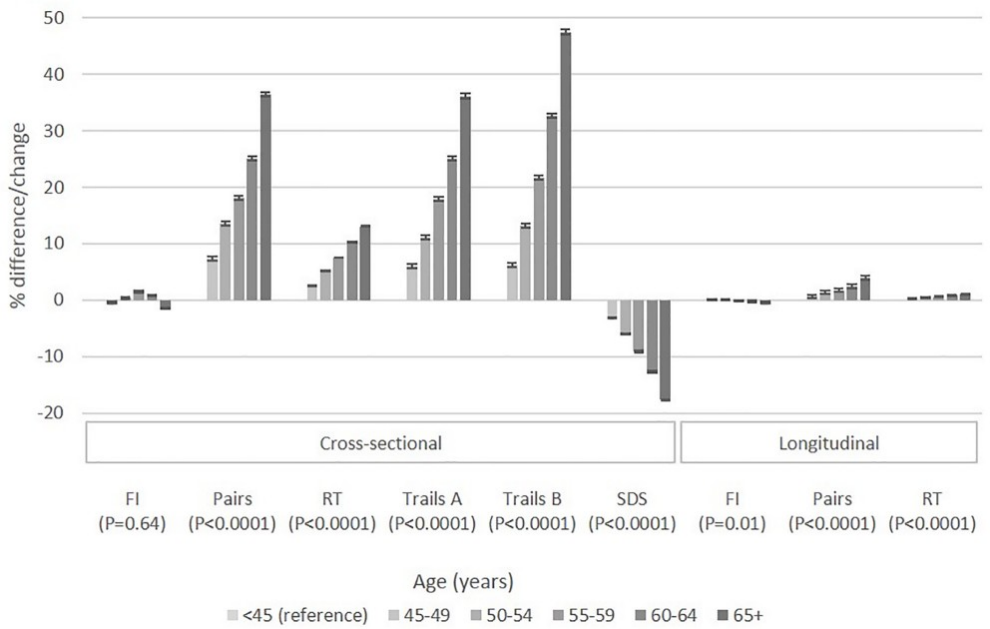
Percentage difference/change in cognition scores with age based on cross-sectional (difference) and longitudinal (change) analysis. To allow comparison across cognitive tests, cognitive difference (cross-sectional) or change (longitudinal) are expressed as a percentage difference/change as a function of the range of each test (See Analyses). Negative % difference/change in FI and SDS represents a lower/decline in performance. Positive % difference/change in Pairs, RT, Trail A and Trail B represents a lower/decline in performance. With increasing age category, Pairs, RT, Trail A, Trail B and SDS significantly changed by 5.9, 2.6, 6.0, 7.8 and 3.4% according to cross-sectional analysis. With increasing age category, Pairs, and RT significantly declined by 1.2 and 3.0% according to longitudinal analysis.

Descriptive statistics across age categories for participants completing at least one of the cognitive function tests at baseline are presented in Table 1. Older individuals were more likely to be current smokers and retired and to have lower income and educational achievement. In multivariable analysis (Table 2, Fig 1), FI scores were higher between ages 55 and 64 and lower at 65+ compared to age <45 (*P*<0.0001). The number of errors made on the Pairs and SDS tests were increasingly higher with older age groups (*P*<0.0001). RT and time taken to complete either Trail test were also increasingly higher with older age (*P*<0.0001). The odds of completing the PM test correctly on the first attempt was lower with older age (*P*<0.0001).

**Table 1:** Baseline characteristics of study population^a^.

Characteristic	Age categories					
	<45 (n = 49,586)	45–49 (n = 63, 062)	50–54 (n = 72,472)	55–59 (n = 85,636)	60–64 (n = 114,183)	65+ (n = 88,684)
Mean (SD) age, years	42.3 (1.3)	47.1 (1.4)	52.1 (1.4)	57.1 (1.4)	61.9 (1.4)	66.9 (1.5)
Female	26703 (53.9)	35199 (55.8)	41252 (56.9)	48055 (56.1)	62550 (54.8)	45394 (51.2)
White	44174 (89.1)	57344 (90.9)	67391 (93.0)	81423 (95.1)	110497 (96.8)	85719 (96.7)
Current smoking	7371 (14.9)	8496 (13.5)	8760 (12.1)	8960 (10.5)	10431 (9.1)	6980 (7.9)
Income						
<18,000	5761 (11.6)	7259 (11.5)	8698 (12.0)	12879(15.0)	26818(23.5)	28297(31.9)
18,000–30,999	8095 (16.3)	10368 (16.4)	12684(17.5)	17772(20.8)	29992(26.3)	23955(27.0)
31,000–51,999	13956 (28.2)	17207 (27.3)	19234(26.5)	21485(25.1)	22689(19.9)	12130(13.7)
52,000–100,000	13289 (26.8)	17056 (27.1)	18687(25.8)	17837(20.8)	12166(10.7)	4669(5.3)
'100,000+	3764 (7.6)	4857 (7.7)	5218(7.2)	4426(5.2)	2958(2.6)	1065(1.2)
will not answer, missing	4721 (9.5)	6315 (10.0)	7951(11.0)	11237(13.1)	19560(17.1)	18568(20.9)
Education, level 4+	34366 (69.3)	42471(67.4)	47811(66.0)	54006(63.1)	63085(55.3)	42024(47.4)
Employment status						
currently employed	43259 (87.2)	55259 (87.6)	61223 (84.5)	61152 (71.4)	43500 (38.1)	11459 (12.9)
retired	70 (0.1)	302 (0.5)	2343 (3.2)	13066 (15.3)	63763 (55.8)	75789 (85.5)
other/not reported	6257 (12.6)	7501 (11.9)	8906 (12.3)	11418 (13.3)	6920 (6.1)	1436 (1.6)
Mean (SD) Townsend deprivation score	-0.7 (3.3)	-1.0 (3.2)	-1.2 (3.1)	-1.4 (3.0)	-1.6 (2.9)	-1.6 (2.9)
Mean (SD) moderate to vigorous physical activity minutes/week	76.5 (96.2)	74.3 (96.0)	71.3 (94.8)	71.2 (95.6)	77.5 (97.2)	80.3 (97.5)
Mean (SD) alcohol drinks/week	1.1 (1.4)	1.2 (1.4)	1.2 (1.5)	1.2 (1.4)	1.2 (1.4)	1.1 (1.4)
Apoe ε4 carriers	11631 (28.5)	15158 (29.1)	16989 (28.5)	19624 (28.2)	26217 (28.2)	20109 (28.2)

^a^Cross-sectional study population. Data drawn from 2006–10. Values are numbers (percentages) unless stated otherwise. All characteristic values are significantly different across age-categories (*P*<0.0001 for all but Apoe: *P* = 0.002).

**Table 2:** Cross-sectional associations between age and cognitive function tests measured at baseline (2006–10).

	Mean (SD) Score	Model 1^a^		Model 2^b^	
		β (95% CI)	*P* value	β (95% CI)	*P* value
^c^Fluid Intelligence (n = 158,673)					
<45	6.02 (2.24)	Reference		Reference	
45–49	5.99 (2.21)	-0.02 (-0.07,0.02)	0.29	-0.05 (-0.09,-0.01)	0.01
50–54	6.10 (2.20)	0.08 (0.04,0.13)	0.0001	0.05 (0.01,0.09)	0.01
55–59	6.22 (2.18)	0.20 (0.16,0.24)	<.0001	0.19 (0.16,0.23)	<.0001
60–64	6.04 (2.12)	0.02 (-0.02,0.06)	0.41	0.11 (0.07,0.15)	<.0001
65+	5.65 (2.03)	-0.38 (-0.42,-0.34)	<.0001	-0.19 (-0.23,-0.14)	<.0001
*Trend*		-0.06 (-0.06,-0.05)	<.0001	0.002 (-0.01,0.01)	0.64
^d^^e^Pairs Matching (n = 461,393)					
<45	1.30 (0.63)	Reference		Reference	
45–49	1.37 (0.63)	0.07 (0.06,0.08)	<.0001	0.07 (0.06,0.08)	<.0001
50–54	1.42 (0.63)	0.12 (0.12,0.13)	<.0001	0.13 (0.12,0.13)	<.0001
55–59	1.46 (0.62)	0.16 (0.16,0.18)	<.0001	0.17 (0.16,0.17)	<.0001
60–64	1.53 (0.62)	0.23 (0.22,0.23)	<.0001	0.22 (0.22,0.23)	<.0001
65+	1.62 (0.61)	0.32 (0.32,0.33)	<.0001	0.31 (0.30,0.32)	<.0001
*Trend*		0.06 (0.06,0.06)	<.0001	0.06 (0.06,0.06)	<.0001
^d^Reaction Time (n = 468,534)					
<45	502.7 (91.3)	Reference		Reference	
45–49	520.1 (96.4)	17.0 (15.8,18.3)	<.0001	17.9 (16.7,19.1)	<.0001
50–54	538.2 (100.6)	34.9 (33.7,36.1)	<.0001	35.9 (34.7,37.1)	<.0001
55–59	555.7 (104.5)	52.6 (51.5,53.8)	<.0001	52.4 (51.3,53.6)	<.0001
60–64	576.8 (109.6)	73.9 (72.8,75.0)	<.0001	71.0 (69.8,72.3)	<.0001
65+	599.0 (115.5)	96.8 (95.6,97.9)	<.0001	90.8 (89.4,92.2)	<.0001
*Trend*		19.4 (19.2,19.6)	<.0001	17.9 (17.7,18.2)	<.0001
^d^^e^Trail A (n = 100,354)					
<45	3.44 (0.31)	Reference		Reference	
45–49	3.50 (0.31)	0.06 (0.05,0.07)	<.0001	0.06 (0.05,0.07)	<.0001
50–54	3.55 (0.31)	0.10 (0.10,0.11)	<.0001	0.11 (0.10,0.11)	<.0001
55–59	3.61 (0.31)	0.17 (0.16,0.18)	<.0001	0.17 (0.16,0.17)	<.0001
60–64	3.68 (0.31)	0.24 (0.23,0.25)	<.0001	0.22 (0.22,0.23)	<.0001
65+	3.77 (0.32)	0.34 (0.33,0.34)	<.0001	0.31 (0.30,0.32)	<.0001
*Trend*		0.07 (0.06,0.07)	<.0001	0.06 (0.06,0.06)	<.0001
^d^^e^Trail B (n = 100,352)					
<45	3.94 (0.31)	Reference		Reference	
45–49	4.00 (0.30)	0.06 (0.05,0.07)	<.0001	0.06 (0.05,0.07)	<.0001
50–54	4.06 (0.31)	0.12 (0.11,0.13)	<.0001	0.12 (0.12,0.13)	<.0001
55–59	4.14 (0.31)	0.20 (0.19,0.21)	<.0001	0.20 (0.19,0.20)	<.0001
60–64	4.23 (0.32)	0.30 (0.29,0.31)	<.0001	0.28 (0.27,0.29)	<.0001
65+	4.35 (0.33)	0.41 (0.40,0.42)	<.0001	0.39 (0.38,0.40)	<.0001
*Trend*		0.08 (0.08,0.08)	<.0001	0.07 (0.07,0.08)	<.0001
^c^Symbol Digit Substitution (n = 114,152)					
<45	23.4 (4.9)	Reference		Reference	
45–49	22.2 (4.7)	-1.2 (-1.3,-1.1)	<.0001	-1.2 (-1.3,-1.1)	<.0001
50–54	21.1 (4.7)	-2.3 (-2.4,-2.3)	<.0001	-2.3 (-2.4,-2.2)	<.0001
55–59	19.8 (4.7)	-3.6 (-3.7,-3.5)	<.0001	-3.6 (-3.7,-3.4)	<.0001
60–64	18.2 (4.6)	-5.2 (-5.3,-5.1)	<.0001	-4.9 (-5.1,-4.8)	<.0001
65+	16.1 (4.8)	-7.3 (-7.4,-7.1)	<.0001	-6.9 (-7.0,-6.8)	<.0001
*Trend*		-1.4 (-1.4,-1.4)	<.0001	-1.3 (-1.3,-1.3)	<.0001
^c^Prospective Memory Test (n = 164,092)					
	% correct	OR (95% CI)	*P* value	OR (95% CI)	*P* value
<45	81.3	Reference		Reference	
45–49	81.1	0.99 (0.94, 1.04)	0.63	0.96 (0.91, 1.01)	0.12
50–54	79.5	0.90 (0.85, 0.94)	<.0001	0.86 (0.81, 0.90)	<.0001
55–59	78.9	0.86 (0.82, 0.90)	<.0001	0.82 (0.78, 0.87)	<.0001
60–64	75.6	0.71 (0.68, 0.74)	<.0001	0.69 (0.66, 0.73)	<.0001
65+	68.9	0.51 (0.49, 0.53)	<.0001	0.51 (0.49, 0.54)	<.0001
*Trend*		0.87 (0.86, 0.88)	<.0001	0.89 (0.88, 0.89)	<.0001

^a^Model 1: adjusted for sex

^b^Model 2: adjusted for sex, smoking, Townsend deprivation index, education, income, alcohol intake, physical activity, ethnicity, and employment status.

^c^Negative beta-coefficients for FI and SDS and OR <1 for PM correspond to lower performance compared to <45.

^d^Positive beta-coefficients for Pairs, RT, Trail A and Trail B correspond to lower performance compared to <45.

^e^Pairs Matching and Trail test scores were log transformed prior to analysis

Overall, men performed better than women on the FI, RT, Pairs, and Trail tests after multivariable adjustment (*P*<0.0001). Women performed better than men on the SDS test (*P*<0.0001). Sex×age interactions were observed for FI, RT, Pairs, and SDS tests (*P*<0.0001, S5 Table). Most notable is the lower FI scores for the 65+ age cohort compared to the <45 age cohort among men (β = -0.32, 2.4% difference) than among women (β = -0.08, 0.64% difference) according to multivariable models only.

Participants with higher educational attainment performed better than those with lower attainment for all examined tests (*P*<0.0001). *APOE* ε4 non-carriers performed significantly better than *APOE* ε4 carriers on the Pairs (*P*<0.0001), SDS (*P*<0.0001), Trail A (*P*<0.0001) and Trail B (*P*<0.0001) tests. Education×age interactions were observed for Trail B (*P*<0.0001) and PM (*P*<0.0001) tests (S6 Table), while significant *APOE* ×age interactions were observed for RT (*P* = 0.001) and SDS (*P*<0.0001) (S7 Table). Interpretations of these interactions were nevertheless unclear when reviewing stratified results and so they may be chance findings, have limited clinical significance or warrant further investigation.

### Longitudinal analysis

Up to 26,005 participants returned for at least one follow-up assessment of FI, Pairs, RT and PM tests. These, but Overall, these participants were more likely white, male, nonsmokers, employed with lower Townsend deprivation indices and reporting higher income, higher educational attainment and lower alcohol consumption compared to those with no follow-up assessments (*P*<0.0001). However, these participants presented with similar *cross-age* characteristics (S8 Table) as those of the full sample (Table 1) and associations between age and cognitive function tests at baseline (S9 Table) were also similar to those of the full sample (Table 2). Mean performance on the FI, Pairs, and PM tests generally *improved* at the first follow-up (~4 years) across age groups, suggesting ‘practice effects’ for these tests. Some UK participants did not complete the first follow-up (~4 years) but returned for the second follow-up (~6 years). Because the mean scores were lower than baseline for these participants, practice effects were likely considerably weaker than that observed for the first follow-up. Table 3 and Fig 1 present results of the longitudinal analysis over an average follow-up time of 5.1 years. In multivariable analysis, FI decreased over follow-up among participants 65+ at baseline compared to those <45 years at baseline (Model 1, *P* = 0.002), but was no longer statistically significant in multivariable analysis once accounting for multiple testing (Model 2, *P* = 0.02). No significant declines were observed for participants aged 45–64 at baseline. RT and Pairs test scores worsened over the follow-up across the older age cohorts compared to the <45 age cohort (*P*<0.0001 for trends). For these two tests, trend tests for age-related declines in performance remained nominally significant when restricting analysis to participants <50 years at baseline and treating age as a continuous variable (*P*<0.05). There was no evidence that age impacted declines in PM test performance (Table 3). When comparing results from longitudinal and cross-sectional analysis (Fig 1), the latter yielded 10 to 12-fold higher changes in RT with age and 8 to 10-fold higher changes in Pairs with age.

**Table 3:** Longitudinal analysis of age and cognitive change.

	Model 1^a^		Model 2^b^	
	β (95% CI)	*P* value	β (95% CI)	*P* value
^c^Fluid Intelligence (n = 7430, 4430, 3813)				
<45	Reference		Reference	
45–49	0.009 (-0.03,0.04)	0.63	0.005 (-0.03,0.04)	0.79
50–54	0.01 (-0.02,0.05)	0.41	0.01 (-0.02,0.04)	0.57
55–59	-0.003 (-0.04,0.03)	0.85	-0.01 (-0.04,0.02)	0.60
60–64	-0.03 (-0.06,0.004)	0.08	-0.02 (-0.06,0.01)	0.18
65+	-0.05 (-0.09,-0.02)	0.002	-0.05 (-0.09,-0.01)	0.02
*Trend*	-0.01 (-0.02,-0.01)	<.0001	-0.01 (-0.02,-0.003)	0.006
^d^^e^Pairs Matching (n = 25371, 18293, 10417)				
<45	Reference		Reference	
45–49	0.01 (-0.0005,0.01)	0.07	0.01 (-0.0001,0.01)	0.05
50–54	0.01 (0.01,0.02)	<.0001	0.01 (0.01,0.02)	<.0001
55–59	0.02 (0.01,0.02)	<.0001	0.02 (0.01,0.02)	<.0001
60–64	0.03 (0.02,0.03)	<.0001	0.02 (0.02,0.03)	<.0001
65+	0.04 (0.03,0.05)	<.0001	0.04 (0.03,0.05)	<.0001
*Trend*	0.01 (0.01,0.01)	<.0001	0.01 (0.01,0.01)	<.0001
^d^Reaction Time (n = 25,739, 18399, 10749)				
<45	Reference		Reference	
45–49	1.29 (0.40,2.18)	0.004	1.38 (0.49,2.27)	0.002
50–54	3.22 (2.37,4.08)	<.0001	3.30 (2.44,4.16)	<.0001
55–59	4.48 (3.67,5.30)	<.0001	4.46 (3.63,5.29)	<.0001
60–64	6.33 (5.53,7.14)	<.0001	6.20 (5.31,7.09)	<.0001
65+	7.68 (6.77,8.59)	<.0001	7.46 (6.41,8.51)	<.0001
*Trend*	1.52 (1.43,1.72)	<.0001	1.58 (1.34,1.70)	<.0001
^c^Prospective Memory Test (n = 7605, 4463, 3979)				
	OR (95% CI)	*P* value	OR (95% CI)	*P* value
<45	Reference		Reference	
45–49	1.01 (0.90, 1.14)	0.94	0.99 (0.88, 1.12)	0.79
50–54	0.95 (0.86, 1.06)	0.38	0.92 (0.82, 1.03)	0.13
55–59	1.01 (0.91, 1.12)	0.92	0.97 (0.87, 1.08)	0.52
60–64	0.94 (0.85, 1.04)	0.27	0.92 (0.82, 1.03)	0.14
65+	0.90 (0.81, 1.00)	0.05	0.88 (0.77, 0.99)	0.04
*Trend*	0.98 (0.96, 1.00)	0.03	0.98 (0.96, 1.00)	0.06

*Note*: Shown are results from linear mixed models with random intercept and time (slope):

^a^Model 1: included time, age, sex, baseline test score, and all possible interactions with time. The time×age interaction term allows the calculation of the yearly rate of decline by age group with reference to the <45 age group.

^b^Model 2: included time, age, sex, baseline test score, smoking, Townsend deprivation index, education, income, alcohol intake, physical activity, ethnicity, employment status, number of follow-up cognitive function tests completed, whether participants completed an on-line cognitive function test prior to the second follow-up (applicable to fluid intelligence and pairs matching tests only), and all possible interactions with time. The time×age interaction term allows the calculation of the yearly rate of decline by age group with reference to the <45 age group.

^c^Negative beta-coefficients for FI and OR <1 for PM correspond to declines in performance compared to <45. Sample sizes (n) correspond to baseline, first and second follow-up, respectively.

^d^Positive beta-coefficients for Pairs and RT correspond to declines in performance compared to <45. Sample sizes (n) correspond to baseline, first and second follow-up, respectively.

^e^Pairs matching scores were log transformed prior to analysis.

The impact of age on FI, RT and Pairs tests were similar when analyses were performed separately for men and women (S10 Table), for lower and higher educational attainment (S11 Table), and for *APOE* ε4 carriers and non-carriers (S12 Table). Significant interactions with baseline score performance were observed for age-specific declines in Pairs (*P*<0.0001) and RT (*P* = 0.002) test performance (S13 Table). Although significant declines were observed in both strata for Pairs and RT, greater declines with age were observed among those with an initially low baseline test score.

We examined attrition and practice effects on FI and PM by comparing the characteristics and mean-level performance in the group of returnees with that of a previously untested group of participants (S1 File). We estimated that practice effects inflated FI and PM scores by up to ~10% and 18%, respectively, and that inflations were greater for older age cohorts.

## Discussion

In the current study of a large cohort of individuals aged 38 to 73 at baseline, we observed significantly lower performance on memory, attention and processing tasks across successive age groups. Reasoning scores, based on the fluid intelligence test, were higher with successive age group until 60, then dropped to less than that of under 45 year olds. Longitudinal analysis of a subset of individuals with repeated measures of four tests showed linear declines in visual memory and processing speed tasks with age but of a much lesser degree than those observed in cross-sectional analyses. Decline rates in reasoning and prospective memory did not significantly differ with age. Taken together, our findings suggest that decline in cognitive abilities before age 65 is evident but small, and that observed cross-sectional differences in cognition from middle to older adult years may be due largely to age cohort effects.

The age at which cognitive decline occurs at the population level has been subject to debate. Estimates from cross-sectional studies reflect the effects of biological ageing but also cohort effects [2]. In the UK Biobank we observed marked differences in educational attainment between the youngest and oldest age groups. Income and employment status were also different but anticipated given life-stage of data collection. Accounting for education and measures of socioeconomic status influenced cross-age cohort differences, particularly those for FI, but not enough to narrow the differences in results from longitudinal analysis. Other factors or more detailed data on education and socioeconomic status likely contribute to the large age-cohort differences observed in the UK Biobank.

While longitudinal data are often considered superior to cross-sectional for characterizing decline in cognitive abilities, they may underestimate the effect of age because of selective sample retention (or attrition) and practice effects [2, 22–24]. Indeed, we observed evidence of both factors in the UK Biobank. The subset of participants undergoing repeat assessments presented with a different and potentially favorable risk factor profile to those providing only baseline measures. While some argue that samples in longitudinal studies are likely to be positively biased (i.e. presenting with less decline) in comparison with the samples in cross-sectional studies, it does not necessarily follow that this would lead to different patterns of age relations unless there is a relationship between the initial level of functioning and the rate of change [3]. In the current study, stratifying on educational attainment did not impact age-differences in baseline cognitive scores or in cognitive decline rates. Age-specific differences in declines for RT and Pairs test performance were observed when considering initial baseline scores; greater declines with age were observed among those with an initially low baseline test score. These findings contrast with those of other, but much smaller, studies which suggest that the age trends in cognitive functioning are parallel across different initial levels [3, 25–29].

Practice effects are defined as improvement on cognitive test performance resulting from learning during repeated testing. The magnitude of these practice effects may be modulated by the length of the test-retest interval; with shorter intervals yielding greater practice effects than longer intervals [3, 30–33]. In the UK Biobank, mean performance on tests generally *improved* at each follow-up, particularly the first, across age groups. The older age groups, nevertheless, appeared to benefit more from repeated testing (S1 File). This may be due to the low initial performance enabling potential for greater improvement compared to the higher initial scores of younger participants. This pattern might also reflect test score limitations at the top of the scale (i.e. ceiling effects).

In the current study we observed a non-linear relationship between age and FI in cross-sectional analysis and a similar but non-significant pattern in longitudinal analysis. Similar observations were reported by Kievet et al [11] who examined only FI in the UK Biobank and using structural equation modeling; results made available while the current study was under-review. Fluid ability is involved in processing novel information efficiently and with increased flexibility [5, 34, 35]. Smaller cross-sectional [8, 36] and longitudinal studies [5, 37, 38] have shown that advancing age is associated with a marked decrease in FI performance. Crystallized abilities rely on over-learned cognitive skills or accumulated knowledge and remain preserved or even improve with age in the cognitively intact [39]. Scores for FI also peak early in adulthood whereas scores for crystallized intelligence peak latter; middle age [8, 40–43]. The pattern of results we observed with FI would better align with those reported for crystallized intelligence. Previous UK Biobank investigators have also preferred the test label ‘verbal-numerical reasoning’ as opposed to the UK Biobank’s label ‘fluid intelligence” since performance on some items (i.e. “which number is the largest”, “age is to years as height is to?”, “relaxed means the opposite of?”) more likely rely on crystallized knowledge” [10, 18, 44–50].

Human and animal studies demonstrate sex differences in cognitive function in adulthood and ageing [6, 51, 52]. For example, on average, men perform better on spatial tasks, and women on verbal tasks [51]. Much of this literature is based on interviewer-based testing whereas tests administered in the UK Biobank were self-administered computer-based and, with the exception of a few fluid intelligence items, captured largely non-verbal tasks. The generally better performance of men over women in the UK Biobank is nevertheless consistent with previous research. However, for fluid intelligence we observed a greater difference in scores with increasing age among men compared to women and only when accounting for confounders. This sex×age interaction was not apparent in longitudinal analysis and further emphasizes that differences between sexes in cognitive *decline* are likely due to cohort differences in social factors rather than biological differences.

Most longitudinal studies covering a wide age range (~20 to 90 years) have had sample sizes less than 2000 but also generally report minimal cognitive decline before the age of 65 (reviewed in [2])[9]. Lundervold et al [9] recently reported a linear age-related decline via cross-sectional and longitudinal analysis with a lower change in the latter but included only 163 participants. Hughes et al [7] examined cognitive decline among ~2,500 participants aged 25 to 95 years at recruitment in the MIDUS study. Multiple cognitive domains were measured by a brief telephone battery and all showed significant but small declines over 9 years, with differences in the timing and extent of change. Processing speed showed the earliest and steepest declines [7]. Our findings generally align with those of a 10 year follow-up of ~7,400 participants aged 45–70 at recruitment of the Whitehall Study [4]. Performance on tests of memory, reasoning and phonemic and semantic fluency were progressively lower with older age categories. In longitudinal analyses, there was some evidence of greater decline at older ages and of a linear trend in decline with increasing age for some of the tests, particularly in men. However, the cross sectional data considerably overestimated cognitive decline among women but not in men, which the authors attributed to cohort differences in education.

The large sample size, age distribution, recency and availability of information on potential confounders are key strengths of the current study. However, a number of limitations should be considered when interpreting the results. The approach to data capture in the UK Biobank aimed to optimize the accuracy and completeness of the data collected, while also maximizing the efficiency of the process. To this end, brief tests of cognition that can be self-administered, are easily repeatable, and have associations with future cognitive decline were selected [53, 54] and further developed in the UK Biobank. Few tests were administered for different cognitive function domains and the tests employed are thus brief, non-standardized and lack external validation. Similarly, whether tests present with floor or ceiling effects has also not been vetted, and should they exist, may vary with age. The testing environment could not be fully standardized due to the self-administrative nature of these tests, particularly those completed at home. Baseline covariates for the web-administered cognitive tests were not collected at the same time as the tests. Taken together, measurement error contributing to type 2 error may be of concern as well as an incomplete representation of cognition. FI and PM tests were administered part way through recruitment and were only used in ten of the recruiting sites. Although power-loss attributable to imprecise measures of cognition may have been compensated for by the large sample size, it is unknown whether measurement error was random or, for example, varied by age or time of recruitment. The UK Biobank is also not representative of the sampling population, with evidence of a ‘healthy volunteer’ selection bias [55] and thus extrapolation of our findings to a more general population is limited. Indeed, this bias was especially evident in the current longitudinal study as demonstrated by characteristic differences between the larger baseline sample and follow-up sample as well as our attrition analysis.

In summary, our findings suggest that declines in cognitive abilities between the end of the fourth decade and age 65 are small. Because the pathophysiological process of dementia may begin years before clinical symptoms [1], the effectiveness of early intervention efforts aiming to treat or delay cognitive decline might only be clinically realized upon older age. Evidence of improving social factors for earlier generations may protect younger individuals from premature cognitive decline.

## Supporting information

S1 File Supplemental materials, methods and notes. (PDF)

S1 Table Assessment center order of operations (Main Protocol). (PDF)

S2 Table Age-stratified sample sizes for cross-sectional analysis. (PDF)

S3 Table Age and follow-up stratified sample sizes for longitudinal analysis. (PDF)

S4 Table Follow-up times (years) from baseline assessment for longitudinal analysis. (PDF)

S5 Table Sex-stratified cross-sectional associations between age and cognitive function tests measured at baseline (2006–10). (PDF)

S6 Table Education-stratified cross-sectional associations between age and cognitive function tests measured at baseline (2006–10). (PDF)

S7 Table Apoe-stratified cross-sectional associations between age and cognitive function tests measured at baseline (2006–10). (PDF)

S8 Table Baseline characteristics of longitudinal study population. (PDF)

S9 Table Cross-sectional associations between age and cognitive function tests measured at baseline (2006–10) for follow-up sample and for full sample. (PDF)

S10 Table Sex-stratified longitudinal analysis of age and cognitive change. (PDF)

S11 Table Education-stratified longitudinal analysis of age and cognitive change. (PDF)

S12 Table Apoe-stratified longitudinal analysis of age and cognitive change. (PDF)

S13 Table Baseline-cognition -stratified longitudinal analysis of age and cognitive change. (PDF)

S14 Table Baseline characteristics of ‘follow-up only’ study population. (PDF)

## References

[1] Sperling RA, Aisen PS, Beckett LA, Bennett DA, Craft S, Fagan AM, et al Toward defining the preclinical stages of Alzheimer’s disease: Recommendations from the National Institute on Aging-Alzheimer’s Association workgroups on diagnostic guidelines for Alzheimer’s disease. Alzheimer’s & dementia: the journal of the Alzheimer’s Association. 2011;7(3):280–92. 10.1016/j.jalz.2011.03.003 PMC3220946 21514248

[2] Rönnlund M, Nyberg L, Bäckman L, Nilsson L-G. Stability, growth, and decline in adult life span development of declarative memory: cross-sectional and longitudinal data from a population-based study. Psychology and aging. 2005;20(1):3 10.1037/0882-7974.20.1.3 15769210

[3] Salthouse TA, Schroeder DH, Ferrer E. Estimating retest effects in longitudinal assessments of cognitive functioning in adults between 18 and 60 years of age. Developmental psychology. 2004;40(5):813 10.1037/0012-1649.40.5.813 15355168

[4] Singh-Manoux A, Kivimaki M, Glymour MM, Elbaz A, Berr C, Ebmeier KP, et al Timing of onset of cognitive decline: results from Whitehall II prospective cohort study. British Medical Journal. 2012;344:d7622 10.1136/bmj.d7622 22223828 PMC3281313

[5] Salthouse TA. When does age-related cognitive decline begin? Neurobiology of aging. 2009;30(4):507–14. 10.1016/j.neurobiolaging.2008.09.023 19231028 PMC2683339

[6] Miller DI, Halpern DF. The new science of cognitive sex differences. Trends in cognitive sciences. 2014;18(1):37–45. 10.1016/j.tics.2013.10.011 . 24246136

[7] Hughes ML, Agrigoroaei S, Jeon M, Bruzzese M, Lachman ME. Change in cognitive performance from midlife into old age: Findings from the Midlife in the United States (MIDUS) study. Journal of the International Neuropsychological Society. 2018:1–16. 10.1017/S1355617718000425 PMC6170692 30019663

[8] Hartshorne JK, Germine LT. When does cognitive functioning peak? The asynchronous rise and fall of different cognitive abilities across the life span. Psychological science. 2015;26(4):433–43. 10.1177/0956797614567339 25770099 PMC4441622

[9] Lundervold AJ, Wollschläger D, Wehling E. Age and sex related changes in episodic memory function in middle aged and older adults. Scandinavian journal of psychology. 2014;55(3):225–32. 10.1111/sjop.12114 24601911 PMC4314696

[10] Hagenaars SP, Harris SE, Davies G, Hill WD, Liewald DC, Ritchie SJ, et al Shared genetic aetiology between cognitive functions and physical and mental health in UK Biobank (N = 112 151) and 24 GWAS consortia. Molecular psychiatry. 2016;21(11):1624–32. 10.1038/mp.2015.225 26809841 PMC5078856

[11] Kievit RA, Fuhrmann D, Borgeest GS, Simpson-Kent IL, Henson RN. The neural determinants of age-related changes in fluid intelligence: a pre-registered, longitudinal analysis in UK Biobank. Wellcome open research. 2018;3. 10.12688/wellcomeopenres.14241.2 PMC5909055 29707655

[12] UK Biobank Coordinating Centre. UK Biobank: Protocol for a large-scale prospective epidemiological resource. 2007.

[13] Cullen B, Nicholl BI, Mackay DF, Martin D, Ul-Haq Z, McIntosh A, et al Cognitive function and lifetime features of depression and bipolar disorder in a large population sample: Cross-sectional study of 143,828 UK Biobank participants. Eur Psychiatry. 2015;30(8):950–8. 10.1016/j.eurpsy.2015.08.006 . 26647871

[14] UK Biobank Coordinating Centre. Cognitive Function Tests: Assessment Centre 2006 [cited 2018 October]. http://biobank.ctsu.ox.ac.uk/crystal/label.cgi?id=100026.

[15] UK Biobank Coordinating Centre. Cognitive Function Tests: On-line 2014 [cited 2018 October]. http://biobank.ctsu.ox.ac.uk/crystal/label.cgi?id=116.

[16] McDaniel MA, Scullin MK. Implementation intention encoding does not automatize prospective memory responding. Memory & Cognition. 2010;38(2):221–32. 20173194 10.3758/MC.38.2.221

[17] Cleutjens FA, Spruit MA, Ponds RW, Dijkstra JB, Franssen FM, Wouters EF, et al Cognitive functioning in obstructive lung disease: results from the United Kingdom biobank. Journal of the American Medical Directors Association. 2014;15(3):214–9. 10.1016/j.jamda.2013.12.007 24513227

[18] Lyall DM, Cullen B, Allerhand M, Smith DJ, Mackay D, Evans J, et al Cognitive test scores in UK Biobank: data reduction in 480,416 participants and longitudinal stability in 20,346 participants. PloS one. 2016;11(4):e0154222 10.1371/journal.pone.0154222 27110937 PMC4844168

[19] UK Biobank Coordinating Centre. UK Biobank touch-screen questionnaire: final version. 2006.

[20] Bycroft C, Freeman C, Petkova D, Band G, Elliott LT, Sharp K, et al. Genome-wide genetic data on~ 500,000 UK Biobank participants. bioRxiv. 2017:166298.

[21] Laird NM, Ware JH. Random-effects models for longitudinal data. Biometrics. 1982;38(4):963–74. . 7168798

[22] Schaie KW. Internal validity threats in studies of adult cognitive development. Cognitive development in adulthood: Progress in cognitive development research. 1988:241–72.

[23] Rabbitt P, Diggle P, Smith D, Holland F, Mc Innes L. Identifying and separating the effects of practice and of cognitive ageing during a large longitudinal study of elderly community residents. Neuropsychologia. 2001;39(5):532–43. 11254936 10.1016/s0028-3932(00)00099-3

[24] Euser SM, Schram MT, Hofman A, Westendorp RG, Breteler MM. Measuring cognitive function with age: the influence of selection by health and survival. Epidemiology. 2008;19(3):440–7. 10.1097/EDE.0b013e31816a1d31 18414086

[25] Christensen H, Henderson A. Is age kinder to the initially more able? A study of eminent scientists and academics. Psychological medicine. 1991;21(4):935–46. 1780406 10.1017/s0033291700029925

[26] Christensen H, Hofer SM, MacKinnon AJ, Korten AE, Jorm AF, Henderson AS. Age is no kinder to the better educated: absence of an association investigated using latent growth techniques in a community sample. Psychological medicine. 2001;31(1):15–28. 11200953 10.1017/s0033291799002834

[27] Deary IJ, Starr JM, MacLennan WJ. Is age kinder to the initially more able?: Differential ageing of a verbal ability in the Healthy Old People in Edinburgh Study. Intelligence. 1998;26(4):357–75.

[28] Owens WA. Is age kinder to the initially more able? Journal of gerontology. 1959. 10.1093/geronj/14.3.334 14429568

[29] Sliwinski M, Buschke H. Cross-sectional and longitudinal relationships among age, cognition, and processing speed. Psychology and aging. 1999;14(1):18 10224629 10.1037//0882-7974.14.1.18

[30] Hultsch DF, Hertzog C, Small BJ, McDonald-Miszczak L, Dixon RA. Short-term longitudinal change in cognitive performance in later life. Psychology and aging. 1992;7(4):571 1466826 10.1037//0882-7974.7.4.571

[31] Bäckman L, Nilsson L-G. Semantic memory functioning across the adult life span. European Psychologist. 1996;1(1):27–33.

[32] Schaie KW. Intellectual development in adulthood: The Seattle longitudinal study: Cambridge University Press; 1996.

[33] Salthouse TA. Effects of age and ability on components of cognitive change. Intelligence. 2013;41(5):501–11. 10.1016/j.intell.2013.07.005 24159248 PMC3804359

[34] Hess TM. Memory and aging in context. Psychological bulletin. 2005;131(3):383 10.1037/0033-2909.131.3.383 15869334

[35] Horn JL, Cattell RB. Refinement and test of the theory of fluid and crystallized general intelligences. Journal of Educational Psychology. 1966;57(5):253–70. 5918295 10.1037/h0023816

[36] Kievit RA, Davis SW, Griffiths J, Correia MM, Henson RN. A watershed model of individual differences in fluid intelligence. Neuropsychologia. 2016;91:186–98. 10.1016/j.neuropsychologia.2016.08.008 27520470 PMC5081064

[37] Ghisletta P, Rabbitt P, Lunn M, Lindenberger U. Two thirds of the age-based changes in fluid and crystallized intelligence, perceptual speed, and memory in adulthood are shared. Intelligence. 2012;40(3):260–8.

[38] Schaie KW. The course of adult intellectual development. American psychologist. 1994;49(4):304 8203802 10.1037//0003-066x.49.4.304

[39] Christensen H. What cognitive changes can be expected with normal ageing? Australian and New Zealand Journal of Psychiatry. 2001;35(6):768–75. 10.1046/j.1440-1614.2001.00966.x 11990887

[40] Bayley N. Development of mental abilities. Carmichael’s manual of child psychology. 1970;1:1163–209.

[41] Doppelt JE, Wallace WL. Standardization of the Wechsler adult intelligence scale for older persons. The Journal of Abnormal and Social Psychology. 1955;51(2):312. 10.1037/h0044391 13263050

[42] Fox C, Birren JE. Some factors affecting vocabulary size in later maturity: Age, education, and length of institutionalization. Journal of gerontology. 1949;4(1):19–26. 18125187 10.1093/geronj/4.1.19

[43] Sorenson H. Mental ability over a wide range of adult ages. Journal of Applied Psychology. 1933;17(6):729.

[44] Davies G, Lam M, Harris SE, Trampush JW, Luciano M, Hill WD, et al Study of 300,486 individuals identifies 148 independent genetic loci influencing general cognitive function. Nature communications. 2018;9(1):2098 10.1038/s41467-018-04362-x 29844566 PMC5974083

[45] Shen X, Cox SR, Adams MJ, Howard DM, Lawrie SM, Ritchie SJ, et al Resting-State Connectivity and Its Association With Cognitive Performance, Educational Attainment, and Household Income in the UK Biobank. Biological Psychiatry: Cognitive Neuroscience and Neuroimaging. 2018. 10.1016/j.bpsc.2018.06.007 PMC6289224 30093342

[46] Cullen B, Newby D, Lee D, Lyall DM, Nevado-Holgado AJ, Evans JJ, et al Cross-sectional and longitudinal analyses of outdoor air pollution exposure and cognitive function in UK Biobank. Scientific reports. 2018;8(1):12089 10.1038/s41598-018-30568-6 30108252 PMC6092329

[47] Hill WD, Davies G, Harris S, Hagenaars S, Davies G, Deary IJ, et al Molecular genetic aetiology of general cognitive function is enriched in evolutionarily conserved regions. Translational psychiatry. 2016;6(12):e980 10.1038/tp.2016.246 27959336 PMC5290340

[48] Hagenaars SP, Gale CR, Deary IJ, Harris SE. Cognitive ability and physical health: a Mendelian randomization study. Scientific reports. 2017;7(1):2651 10.1038/s41598-017-02837-3 28572633 PMC5453939

[49] Nevado-Holgado AJ, Kim C-H, Winchester L, Gallacher J, Lovestone S. Commonly prescribed drugs associate with cognitive function: a cross-sectional study in UK Biobank. BMJ open. 2016;6(11):e012177 10.1136/bmjopen-2016-012177 27903560 PMC5168501

[50] Ge T, Chen CY, Doyle AE, Vettermann R, Tuominen LJ, Holt DJ, et al The Shared Genetic Basis of Educational Attainment and Cerebral Cortical Morphology. Cereb Cortex. 2018 Epub 2018/10/03. 10.1093/cercor/bhy216 30272126 PMC6644848

[51] Li R, Singh M. Sex differences in cognitive impairment and Alzheimer’s disease. Frontiers in neuroendocrinology. 2014;35(3):385–403. 10.1016/j.yfrne.2014.01.002 24434111 PMC4087048

[52] Ingalhalikar M, Smith A, Parker D, Satterthwaite TD, Elliott MA, Ruparel K, et al Sex differences in the structural connectome of the human brain. Proc Natl Acad Sci U S A. 2014;111(2):823–8. 10.1073/pnas.1316909110 24297904 PMC3896179

[53] Brayne C, Day N, Gill C. Methodological issues in screening for dementia. Neuroepidemiology. 1992;11(Suppl. 1):88–93. 1603257 10.1159/000110997

[54] De Jager C, Blackwell AD, Budge MM, Sahakian BJ. Predicting cognitive decline in healthy older adults. The American Journal of Geriatric Psychiatry. 2005;13(8):735–40. 10.1176/appi.ajgp.13.8.735 16085791

[55] Fry A, Littlejohns TJ, Sudlow C, Doherty N, Adamska L, Sprosen T, et al Comparison of sociodemographic and health-related characteristics of UK biobank participants with those of the general population. American journal of epidemiology. 2017;186(9):1026–34. 10.1093/aje/kwx246 28641372 PMC5860371

