# Supplementary material for: Age and cognitive decline in the UK Biobank
Source: PLoS One. 2019 Mar 18;14(3):e0213948. doi: 10.1371/journal.pone.0213948 (PMC6422276; doi:10.1371/journal.pone.0213948)
Supplement: S1 File — (PDF) [file pone.0213948.s001.pdf]

SUPPLEMENTARY METHOD

Participants

UK Biobank investigators sent postal invitations to approximately 9.2 million individuals registered with the UK's National Health Service who were aged 40–69 years and lived within approximately 40 km of one of 22 assessment centers located throughout England, Wales, and Scotland[1]. The age group was targeted because it allows investigation of the common causes of morbidity and premature mortality, and also allows ascertainment of events at an age where such cause-specific outcomes are generally well recorded, with less co-morbidity than outcomes at older ages. About 3 weeks after mailing the invitation letter, people who did not respond were sent a re-invitation letter once only. Personal invitations were paralleled with increasing local awareness of UK Biobank[1]. Between 2006 and 2010, over 502,633 participants aged 37-73 years (5.4% participation rate) provided full informed consent to participate in UK Biobank and completed a 90-minute assessment that included i) touchscreen questionnaires on sociodemographic factors, lifestyle and medical history ii) an in-person interview and iii) physical assessment: (see S1 Table for order of operations).

Measures and procedures

UK Biobank aims to assess the relevance of a wide range of exposures to a wide range of health-related outcomes. The baseline questions and measurements have therefore been chosen carefully to allow this wide assessment to be conducted in the whole cohort. The comprehension and acceptability of each question, the time taken to complete each of them, and their response distributions were examined in pilot studies, which aided the final selection and presentation of suitable questions. The UK Biobank questionnaire was administered in two sequential parts during the assessment center visit: a touch-screen self-completed questionnaire followed by a computer-assisted personal interview (CAPI). Topic areas and questions considered of an exploratory nature have been restricted to the self-completed questionnaire (wherever possible), and questions that needed to be asked by an interviewer required greater evidence of their value to be included. Due to the large size of the UK Biobank cohort, the approach to data capture aimed to optimize the accuracy and completeness of the data collected, while also maximizing the efficiency of the process. Computerized direct

data entry methods were selected in preference to conventional paper questionnaires as these allow internal consistency checks, automated coding, immediate access, and ongoing monitoring and audit. A pre-visit aide memoire is provided to participants prior to attending the assessment center so that they can note certain information (e.g. medications, operations, family history, and birth details) that may be difficult or time-consuming for them to recall during the visit. Pre-coded lists of diseases, drugs, and occupations are built into the CAPI system, along with structured search facilities, to help this information to be recorded (and automatically coded) both rapidly and completely. Data for the current analysis was downloaded in 2017.

Cognitive function testing [2]

Cognitive functioning was assessed using a 15-min computerized battery which was developed specifically for the UK Biobank study to enable population-scale cognitive testing that could be administered without researcher supervision. The battery of tests typically employed to assess cognition are time-consuming and generally unsuitable for self-administration. In addition, they have typically only been administered and validated in much smaller and older populations than in UK Biobank[1]. Following wide consultation, a comprehensive review was conducted of brief tests of cognition that can be self-administered, are easily repeatable within a larger cognitive screening battery [3], and have associations with future cognitive decline. Based on this review, paired associated learning questions to assess global cognition [4] and reaction time tests for touch-screen administration have been developed and refined through piloting to ensure that they provide wide response distributions[1]. A detailed description of all tests are provided on-line [5, 6] and summarized in sections below. Tests administered at the assessment center and in the following order include: Prospective memory (Part 1), Pairs matching, Fluid intelligence, Reaction time and Prospective memory (Part 2). Beginning in 2014, the following tests were completed on-line and at home: Fluid intelligence (excluded from current analysis), Trail Making Test A, Trail Making Test B, Symbol Digit substitution and then Pairs matching (excluded from current analysis). A digit recall test was only performed in a subset of the participants at

baseline and was phased out during the latter part of recruitment and continued on-line. This test was not included for analysis given the different environments in which the tests were completed.

Prospective Memory (PM) Test: This test was added part-way through the baseline assessment period and completed at the assessment centers. Participants were given the following instructions: 'At the end of the games we will show you four colored symbols and ask you to touch the blue square. However, to test your memory, we want you to actually touch the Orange Circle instead'. Participants were scored as zero or one, depending on whether they completed the task on first attempt or not. This test assesses PM: the ability to carry out future intentions at a specific time or in response to a specific event and therefore includes a prospective component (remembering to remember) and a retrospective component (remembering the content of what is to be remembered)[7, 8].

Pairs Matching (Pairs) Test: This episodic visual memory test was completed at the assessment centers. Participants were shown 6 pairs of cards for 5 seconds, which were then turned over. Participants were instructed to select, from recall and in the fewest number of attempts, the pairs of cards that had matching symbols. There was no time limit and the participants could make as many attempts as they needed to find all the pairs. The memory test score in the present study is the total number of errors made during this task until the six pairs of identical cards were touched consecutively. We restricted our analyses to individuals who finished the test and log (+1) transformed the number of errors for the analysis. Transformation was necessary since the distribution of raw scores were zero-inflated and highly skewed to the right. During the pilot phase of the UK Biobank, a subset of participants completed this test twice in immediate succession and the intraclass correlation was 0.17[9].

Fluid Intelligence (FI) Test: This test was added part-way through the baseline assessment period and completed at the assessment centers. Participants were presented with 13 verbal logic/reasoning-type multiple choice questions and had to answer as many as they could within 2 minutes. There were six verbal items and seven numerical items, involving sequence recognition and arithmetic. Incorrect or unattempted questions were scored as zero. The total number of correct answers (max 13) was normally distributed and used for our

analysis. The Cronbach alpha coefficient for these items has been reported elsewhere as 0.62[10]. The UK Biobank describes FI as the "capacity to solve problems that require logic and reasoning ability, independent of acquired knowledge" [5]. Others have preferred the test label 'verbal-numerical reasoning' as opposed to the UK Biobank's label 'fluid Intelligence" since performance on some items more likely rely on crystalized knowledge" [9-17].

Reaction Time (RT) Test: For this measure of simple processing speed, participants completed a timed test of symbol matching at the assessment centers. Participants were shown one pair of cards out of a set of 12 pairs. If both cards displayed a matching symbol, participants pressed a response button as quickly as possible using their dominant hand. Symbols included: equals-sign "=", 3 overlapping triangles, hollow circle, hollow square, ruby-post "H", smiley face, solid circle, solid square, triangle and cross "+". Five 'training' trials were administered, followed by seven test trials. The score for analysis was the mean time (in milliseconds) to press the button, derived from the four trials in which a matching pair occurred. The UK Biobank indicates that i) times under 50 ms must be due to anticipation rather than reaction and so were excluded and ii) times over 2000 ms are ignored since cards had disappeared by then. For the current analysis additional potential outliers were truncated (not excluded) to 100 (min, N=2) or 1000 (max, N=2749) ms as they scored more than 4 SD away from the mean. This approach generated better normally distributed scores than log-transformed scores. However, results were similar when log-transformed values were analyzed (data not shown). Cronbach's alpha for this task has previously been reported as 0.85 [10].

Symbol Digit Substitution (SDS) Test: This test for complex processing speed was completed at follow-up (~2014) on home computers and involves matching numbers to a set of symbols. Participants are presented with symbols paired with digits and are asked to enter the digits that are paired with the symbols in the empty spaces. We used the number of correct substitutions for our analyses. Participants (n=236) who scored 0 or above 40 were truncated to 1 (min) or 40 (max) correct substitutions, respectively, as they scored more than 4 SD away from the mean.

Trail Making Test A and B: These visual attention tests (or executive function) provide information on visual search, scanning, speed of processing, mental flexibility, and executive functions and were completed at follow-up on home computers (~2014). Participants were asked to connect scattered circles containing a sequence of numbers (Trail A) and then to connect circles containing numbers or letters by alternating between them in ascending sequence (Trail B). We used the time taken to complete these tests for our analyses, and these data were log-transformed to account for the right-skewed distribution of the raw scores.

Long-term follow-up and Re-assessment:

Permission was obtained at enrolment from all participants to access all of their past and future medical and other health-related records. This information will supplement information recorded at enrollment and provide follow-up information related to cause-specific mortality and other health events. In addition, and pertinent to the current analysis, subsets of participants completing the baseline visit returned to the assessment centers for up to two follow-up assessments. The first follow-up took place 2012-13 and included 20,346 participants (25,000 invited), all living within 35 km of the Stockport Biobank coordinating center. The same region is currently being targeted for a second follow-up which began in 2014 and also involves an imaging component. At the time of the current study, data from 11,923 participants was available. At follow-up visits participants were invited to repeat the PM, FI, Pairs and RT tests. Longitudinal analysis for the current study were restricted to individuals completing the baseline tests and at least one follow-up test. Thus, longitudinal analysis was restricted to individuals from the Stockport Biobank area.

Although over 500,000 participants took part in the UK Biobank, only those who had data for at least one cognitive function test at the baseline assessment was included for the current analysis. Because some cognitive tasks were added at different stages of baseline assessment or not until 2014 the number of participants varies across tests (Table S2-S3). Beginning 2014, participants were also invited to complete the FI and Pairs tests on home computers. This version of the FI test included an additional question and thus the maximum score was 14. The online FI and Pairs tests were performed before (up to 2 years) or after (up to 0.8 years) the second assessment center follow-up. Among those completing the online and second follow-up test,

mean performance on these tests were lower than any assessment center test (i.e. baseline, follow-up 1 or follow-up 2), regardless of whether they were performed before or after the second assessment center follow-up test, or whether a baseline test was completed. Recall that these tests were introduced at the assessment center part way through recruitment. Differences between on-line and assessment center version of these tests likely arise from the testing environment but nevertheless warranted concern and thus were not included for the current analysis.

SUPPLEMENTARY NOTE

We investigated attrition and practice effects in the longitudinal analysis using a similar approach as that described by Schaie [18] and Ronnlund et al [19]. For each age cohort and cognitive test, we estimated attrition on the first follow-up in the longitudinal sample by subtracting the cross-sectional sample test mean at baseline from the longitudinal sample. Attrition effects are presented as a percent change (i.e. (longitudinal mean – cross-sectional mean)/longitudinal mean)×100. We estimated the mean-level impact of practice effects on longitudinal results for FI and PM tests (tests introduced part way through baseline assessment) leveraging a new sample (N ≤ 14,460) from the target population that did not complete baseline year cognitive tests (i.e. excluded from our primary analysis) but completed the first follow-up (when baseline completers were re-tested). S14 Table present characteristics of the new sample; which were significantly different from the full baseline sample but not significantly different from the longitudinal sample. For each age cohort and cognitive test, the mean test score for the longitudinal sample was subtracted by the mean test score of the new sample. Since the latter shared more characteristics with the longitudinal sample than the cross-sectional sample the difference between scores may be assumed to result from practice effects and not the joint effects of practice and attrition (as reported by Ronnlund et al [19], whose new sample was similar to the cross-sectional sample). However, to further account for sample differences we calculated least squared means (lsmean); adjusting for covariates listed for the multivariable analysis of the cross-sectional sample (see Statistical Analysis of main paper). Practice effects are presented as a percent change (i.e. (longitudinal lsmean – new lsmean)/longitudinal

lsmean)×100. Using this estimate of practice effects we applied a correction to individual's follow-up scores [20]: 1- (practice effect %/100), and re-analyzed the data.

Based on the (crude) means score differences between the re-tested sample and new sample, we estimated that practice effects inflated FI scores by 6.8, 4.2, 7.8, 6.9, 9.7, and 9.2% for <45, 45-49, 50-54, 55-59, 60-64, and 65+, respectively. When FI scores were multivariable adjusted, % change in least square means scores were 6.2, 4.7, 7.6, 6.4, 9.3, and 8.6% and these were used to correct for practice effects. Compared to <45, effect coefficients (P-value) for 45-49, 50-54, 55-59, 60-64, and 65+ were 0.006 (P=0.72), -0.006 (P=0.73), -0.02 (0.32), -0.06 (0.0007), and -0.08 (P<0.0001), respectively, with an overall trend effect of -0.02 (P<0.0001). Expressing these results as % change from baseline equates to 0.05%, -0.04%,-0.12%, -0.45%, and -0.64%, respectively, with an overall trend change of -0.13% (compare these to results presented in Table 3 and Figure 1). Corresponding inflations for the PM test were estimated as 6.5, 8.0, 8.0, 8.0, 10.9, and 18.1%. No attempt was made to perform a longitudinal analysis correcting for practice effects given the binary outcome of this test.

References

1. UK Biobank Coordinating Centre. UK Biobank: Protocol for a large-scale prospective epidemiological resource. 2007.
2. Cullen B, Nicholl BI, Mackay DF, Martin D, Ul-Haq Z, McIntosh A, et al. Cognitive function and lifetime features of depression and bipolar disorder in a large population sample: Cross-sectional study of 143,828 UK Biobank participants. Eur Psychiatry. 2015;30(8):950-8. doi: 10.1016/j.eurpsy.2015.08.006. PubMed PMID: 26647871.
3. Brayne C, Day N, Gill C. Methodological issues in screening for dementia. Neuroepidemiology. 1992;11(Suppl. 1):88-93.
4. De Jager C, Blackwell AD, Budge MM, Sahakian BJ. Predicting cognitive decline in healthy older adults. The American Journal of Geriatric Psychiatry. 2005;13(8):735-40.

5. UK Biobank Coordinating Centre. Cognitive Function Tests: Assessment Centre 2006 [cited 2018 October]. Available from: http://biobank.ctsu.ox.ac.uk/crystal/label.cgi?id=100026.
6. UK Biobank Coordinating Centre. Cognitive Function Tests: On-line 2014 [cited 2018 October]. Available from: http://biobank.ctsu.ox.ac.uk/crystal/label.cgi?id=116.
7. McDaniel MA, Scullin MK. Implementation intention encoding does not automatize prospective memory responding. Memory & Cognition. 2010;38(2):221-32.
8. Cleutjens FA, Spruit MA, Ponds RW, Dijkstra JB, Franssen FM, Wouters EF, et al. Cognitive functioning in obstructive lung disease: results from the United Kingdom biobank. Journal of the American Medical Directors Association. 2014;15(3):214-9.
9. Lyall DM, Cullen B, Allerhand M, Smith DJ, Mackay D, Evans J, et al. Cognitive test scores in UK Biobank: data reduction in 480,416 participants and longitudinal stability in 20,346 participants. PloS one. 2016;11(4):e0154222.
10. Hagenaars SP, Harris SE, Davies G, Hill WD, Liewald DC, Ritchie SJ, et al. Shared genetic aetiology between cognitive functions and physical and mental health in UK Biobank (N=112 151) and 24 GWAS consortia. Molecular psychiatry. 2016;21(11):1624-32. doi: 10.1038/mp.2015.225. PubMed PMID: 26809841; PubMed Central PMCID: PMC5078856 interest.
11. Davies G, Lam M, Harris SE, Trampush JW, Luciano M, Hill WD, et al. Study of 300,486 individuals identifies 148 independent genetic loci influencing general cognitive function. Nature communications. 2018;9(1):2098.
12. Shen X, Cox SR, Adams MJ, Howard DM, Lawrie SM, Ritchie SJ, et al. Resting-State Connectivity and Its Association With Cognitive Performance, Educational Attainment, and Household Income in the UK Biobank. Biological Psychiatry: Cognitive Neuroscience and Neuroimaging. 2018.
13. Cullen B, Newby D, Lee D, Lyall DM, Nevado-Holgado AJ, Evans JJ, et al. Cross-sectional and longitudinal analyses of outdoor air pollution exposure and cognitive function in UK Biobank. Scientific reports. 2018;8(1):12089.

14. Hill WD, Davies G, Harris S, Hagenaars S, Davies G, Deary IJ, et al. Molecular genetic aetiology of general cognitive function is enriched in evolutionarily conserved regions. Translational psychiatry. 2016;6(12):e980.

15. Hagenaars SP, Gale CR, Deary IJ, Harris SE. Cognitive ability and physical health: a Mendelian randomization study. Scientific reports. 2017;7(1):2651.

16. Nevado-Holgado AJ, Kim C-H, Winchester L, Gallacher J, Lovestone S. Commonly prescribed drugs associate with cognitive function: a cross-sectional study in UK Biobank. BMJ open. 2016;6(11):e012177.

17. Ge T, Chen CY, Doyle AE, Vettermann R, Tuominen LJ, Holt DJ, et al. The Shared Genetic Basis of Educational Attainment and Cerebral Cortical Morphology. Cereb Cortex. 2018. Epub 2018/10/03. doi: 10.1093/cercor/bhy216. PubMed PMID: 30272126.

18. Schaie KW. Internal validity threats in studies of adult cognitive development. Cognitive development in adulthood: Progress in cognitive development research. 1988:241-72.

19. Rönnlund M, Nyberg L, Bäckman L, Nilsson L-G. Stability, growth, and decline in adult life span development of declarative memory: cross-sectional and longitudinal data from a population-based study. Psychology and aging. 2005;20(1):3.

20. Marley CJ, Sinnott A, Hall JE, Morris-Stiff G, Woodsford PV, Lewis MH, et al. Failure to account for practice effects leads to clinical misinterpretation of cognitive outcome following carotid endarterectomy. Physiological Reports. 2017;5(11):e13264.
