# Supplementary material for: Age and cognitive decline in the UK Biobank
Source: PLoS One. 2019 Mar 18;14(3):e0213948. doi: 10.1371/journal.pone.0213948 (PMC6422276; doi:10.1371/journal.pone.0213948)
Supplement: S1 Table — (PDF) [file pone.0213948.s002.pdf]

Table S1. Assessment Center Order of Operations (Main Protocol [1])

**Visit station**	**Assessments undertaken**
Reception	• Welcome & registration<br>• Consent
Questionnaire	• Touch screen questionnaire (sociodemographic factors, lifestyle and medical history)<br>• (A) Cognitive function tests<br>1: Prospective Memory: Shape – Part 1<br>2: Pairs matching test<br>3: Fluid intelligence<br>4: Reaction time- Snap<br>1: Prospective Memory: Shape – Part 2
Interview (& blood pressure)	• Interviewer questionnaires<br>• Blood pressure measurement
Physical measurements	• Anthropometrics, hand-grip strength, heel bone ultrasound, spirometry
Sample collection (& exit)	• Blood sample collected<br>• Urine sample sought<br>• Consent & result summary printed<br>• Travel expense claim provided
