# Supplementary material for: Age and cognitive decline in the UK Biobank
Source: PLoS One. 2019 Mar 18;14(3):e0213948. doi: 10.1371/journal.pone.0213948 (PMC6422276; doi:10.1371/journal.pone.0213948)
Supplement: S2 Table — (PDF) [file pone.0213948.s003.pdf]

Table S2. Age-stratified sample sizes for cross-sectional analysis

Age Category	Fluid Intelligence	Pairs Matching	Reaction Time	Trail A	Trail B	Symbol Digit Substitution	Prospective Memory Test
<45	16743	48663	49118	10320	10320	11399	17205
45-49	20254	61920	62480	13896	13896	15287	20825
50-54	23454	71025	71755	16896	16896	18713	24112
55-59	27406	83731	84752	20809	20807	23557	28229
60-64	39808	111083	113010	24639	24639	28419	41146
65+	31008	84971	87419	13794	13794	16777	32575
