# Supplementary material for: Age and cognitive decline in the UK Biobank
Source: PLoS One. 2019 Mar 18;14(3):e0213948. doi: 10.1371/journal.pone.0213948 (PMC6422276; doi:10.1371/journal.pone.0213948)
Supplement: S3 Table — (PDF) [file pone.0213948.s004.pdf]

Table S3. Age and follow-up stratified sample sizes for longitudinal analysis

Age Category	Fluid Intelligence			Pairs Matching			Reaction Time			Prospective Memory		
	baseline	FU1	FU2	baseline	FU1	FU2	baseline	FU1	FU2	baseline	FU1	FU2
<45	551	259	352	2222	1426	1142	2238	1428	1158	559	260	360
45-49	853	420	518	3146	2001	1556	3166	1999	1578	870	420	537
50-54	1177	626	674	4017	2678	1882	4037	2683	1911	1192	628	690
55-59	1622	969	862	5656	4117	2375	5714	4131	2436	1648	977	886
60-64	2040	1334	924	6688	5167	2348	6829	5210	2461	2101	1348	977
65+	1187	822	483	3642	2904	1114	3755	2948	1205	1235	830	529

FU: follow-up
