# Supplementary material for: Age and cognitive decline in the UK Biobank
Source: PLoS One. 2019 Mar 18;14(3):e0213948. doi: 10.1371/journal.pone.0213948 (PMC6422276; doi:10.1371/journal.pone.0213948)
Supplement: S4 Table — (PDF) [file pone.0213948.s005.pdf]

Table S4. Follow-up times (years) from baseline assessment for longitudinal analysis

Age Category	FU	Fluid Intelligence			Pairs Matching			Reaction Time			Prospective Memory		
		Mean	Min	Max	Mean	Min	Max	Mean	Min	Max	Mean	Min	Max
All	FU1	3.0	2.1	3.9	4.3	2.1	6.1	4.3	2.1	6.1	3.0	2.1	3.9
	FU2	5.8	3.8	7.3	6.6	3.8	9.5	6.6	3.8	9.5	5.8	3.8	7.3
<45	FU1	3.1	2.2	3.9	4.5	2.2	6.1	4.5	2.2	6.1	3.1	2.2	3.9
	FU2	5.9	4.2	7.2	6.8	4.2	9.5	6.8	4.2	9.5	5.9	4.2	7.2
45-49	FU1	3.0	2.2	3.9	4.3	2.2	6.1	4.3	2.2	6.1	3.0	2.2	3.9
	FU2	5.8	3.8	7.2	6.7	3.8	9.3	6.7	3.8	9.3	5.8	3.8	7.2
50-54	FU1	3.0	2.3	3.8	4.3	2.3	6.1	4.3	2.3	6.1	3.0	2.3	3.8
	FU2	5.9	4.3	7.3	6.7	4.3	9.4	6.7	4.3	9.4	5.9	4.3	7.3
55-59	FU1	3.0	2.1	3.8	4.3	2.1	6.1	4.3	2.1	6.1	3.0	2.1	3.8
	FU2	5.8	4.3	7.2	6.6	4.3	9.5	6.6	4.3	9.5	5.8	4.3	7.2
60-64	FU1	3.0	2.2	3.9	4.2	2.2	6.1	4.2	2.2	6.1	3.0	2.2	3.9
	FU2	5.8	4.3	7.2	6.6	4.3	9.4	6.6	4.3	9.4	5.8	4.3	7.2
65+	FU1	3.0	2.3	3.9	4.2	2.3	6.1	4.2	2.3	6.1	3.0	2.3	3.9
	FU2	5.7	4.3	7.2	6.5	4.3	9.3	6.5	4.3	9.3	5.7	4.3	7.2

FU: follow-up
