# Supplementary material for: Age and cognitive decline in the UK Biobank
Source: PLoS One. 2019 Mar 18;14(3):e0213948. doi: 10.1371/journal.pone.0213948 (PMC6422276; doi:10.1371/journal.pone.0213948)
Supplement: S5 Table — (PDF) [file pone.0213948.s006.pdf]

Table S5. Sex-Stratified Cross-Sectional Associations Between Age and Cognitive Function Tests Measured At Baseline (2006-10)*

	Women (n≤256,278)					Men (n≤212,256)				
	Mean (SD) Score	Model 1†		Model 2‡		Mean (SD) Score	Model 1†		Model 2‡	
		β (SE)	P	β (SE)	P		β (SE)	P	β (SE)	P
**§Fluid Intelligence**										
<45	5.94 (2.16)	Ref.		Ref.		6.12 (2.33)	Ref.		Ref.	
45-49	5.89 (2.15)	-0.05 (0.03)	0.09	-0.08 (0.03)	0.00	6.13 (2.27)	0.01 (0.03)	0.86	-0.02 (0.03)	0.56
50-54	5.98 (2.11)	0.04 (0.03)	0.18	0.03 (0.03)	0.21	6.26 (2.30)	0.14 (0.03)	<.0001	0.07 (0.03)	0.02
55-59	6.11 (2.11)	0.17 (0.03)	<.0001	0.22 (0.03)	<.0001	6.35 (2.27)	0.23 (0.03)	<.0001	0.14 (0.03)	<.0001
60-64	5.90 (2.05)	-0.05 (0.03)	0.08	0.13 (0.03)	<.0001	6.21 (2.20)	0.09 (0.03)	0.00	0.08 (0.03)	0.01
65+	5.54 (1.93)	-0.40 (0.03)	<.0001	-0.08 (0.03)	0.01	5.76 (2.11)	-0.36 (0.03)	<.0001	-0.32 (0.03)	<.0001
Trend		-0.06 (0.00)	<.0001	0.02 (0.01)	<.0001		-0.06 (0.01)	<.0001	-0.02 (0.01)	<.0001
**¶Pairs Matching**										
<45	1.31 (0.62)	Ref.		Ref.		1.28 (0.65)	Ref.		Ref.	
45-49	1.38 (0.61)	0.06 (0.005)	<.0001	0.06 (0.005)	<.0001	1.36 (0.64)	0.08 (0.006)	<.0001	0.08 (0.006)	<.0001
50-54	1.43 (0.62)	0.12 (0.005)	<.0001	0.12 (0.005)	<.0001	1.41 (0.64)	0.13 (0.006)	<.0001	0.14 (0.006)	<.0001
55-59	1.47 (0.61)	0.16 (0.005)	<.0001	0.16 (0.005)	<.0001	1.45 (0.63)	0.17 (0.005)	<.0001	0.18 (0.005)	<.0001
60-64	1.54 (0.61)	0.22 (0.005)	<.0001	0.21 (0.005)	<.0001	1.51 (0.63)	0.23 (0.005)	<.0001	0.24 (0.005)	<.0001
65+	1.62 (0.60)	0.31 (0.005)	<.0001	0.29 (0.006)	<.0001	1.62 (0.62)	0.34 (0.005)	<.0001	0.34 (0.006)	<.0001
Trend		0.06 (0.001)	<.0001	0.05 (0.001)	<.0001		0.06 (0.001)	<.0001	0.06 (0.001)	<.0001
**¶Reaction Time**										
<45	507.1 (89.8)	Ref.		Ref.		497.6 (92.8)	Ref.		Ref.	
45-49	525.7 (95.7)	18.6 (0.85)	<.0001	19.5 (0.85)	<.0001	513.1 (96.9)	15.5 (0.9)	<.0001	16.0 (0.9)	<.0001
50-54	545.2 (100.4)	38.2 (0.83)	<.0001	39.0 (0.82)	<.0001	528.8 (100.2)	31.2 (0.9)	<.0001	32.4 (0.9)	<.0001
55-59	563.8 (104.4)	56.8 (0.80)	<.0001	56.1 (0.81)	<.0001	545.4 (103.8)	47.8 (0.9)	<.0001	48.5 (0.9)	<.0001
60-64	586.8 (109.4)	79.7 (0.77)	<.0001	77.1 (0.88)	<.0001	564.7 (108.6)	67.1 (0.8)	<.0001	64.6 (0.9)	<.0001
65+	610.8 (116.3)	103.8 (0.81)	<.0001	98.2 (1.00)	<.0001	586.6 (113.3)	88.9 (0.9)	<.0001	83.5 (1.0)	<.0001
Trend		20.7 (0.13)	<.0001	19.3 (0.18)	<.0001		17.8 (0.1)	<.0001	16.5 (0.2)	<.0001
**¶Trail A**										
<45	3.47 (0.31)	Ref.		Ref.		3.41 (0.30)	Ref.		Ref.	
45-49	3.52 (0.31)	0.06 (0.005)	<.0001	0.06 (0.005)	<.0001	3.47 (0.30)	0.06 (0.006)	<.0001	0.06 (0.006)	<.0001
50-54	3.57 (0.31)	0.10 (0.005)	<.0001	0.10 (0.005)	<.0001	3.52 (0.30)	0.11 (0.006)	<.0001	0.11 (0.006)	<.0001
55-59	3.64 (0.32)	0.17 (0.005)	<.0001	0.16 (0.005)	<.0001	3.58 (0.30)	0.17 (0.006)	<.0001	0.17 (0.006)	<.0001

60-64	3.71 (0.32)	0.24 (0.005)	<.0001	0.21 (0.006)	<.0001	3.65 (0.30)	0.25 (0.005)	<.0001	0.23 (0.006)	<.0001
65+	3.80 (0.33)	0.34 (0.006)	<.0001	0.30 (0.007)	<.0001	3.75 (0.31)	0.34 (0.006)	<.0001	0.32 (0.007)	<.0001
Trend		0.07 (0.001)	<.0001	0.06 (0.001)	<.0001		0.07 (0.001)	<.0001	0.06 (0.001)	<.0001
**¶Trail B**										
<45	3.95 (0.30)	Ref.		Ref.		3.92 (0.31)	Ref.		Ref.	
45-49	4.01 (0.30)	0.06 (0.005)	<.0001	0.06 (0.005)	<.0001	3.98 (0.30)	0.06 (0.006)	<.0001	0.06 (0.006)	<.0001
50-54	4.08 (0.31)	0.12 (0.005)	<.0001	0.13 (0.005)	<.0001	4.04 (0.31)	0.12 (0.006)	<.0001	0.12 (0.006)	<.0001
55-59	4.16 (0.31)	0.20 (0.005)	<.0001	0.20 (0.005)	<.0001	4.11 (0.31)	0.19 (0.006)	<.0001	0.19 (0.006)	<.0001
60-64	4.25 (0.32)	0.30 (0.005)	<.0001	0.28 (0.006)	<.0001	4.21 (0.31)	0.29 (0.006)	<.0001	0.28 (0.006)	<.0001
65+	4.37 (0.33)	0.42 (0.006)	<.0001	0.39 (0.007)	<.0001	4.32 (0.33)	0.40 (0.006)	<.0001	0.39 (0.007)	<.0001
Trend		0.08 (0.001)	<.0001	0.07 (0.001)	<.0001		0.08 (0.001)	<.0001	0.08 (0.001)	<.0001
**§Symbol Digit Substitution**										
<45	23.5 (4.9)	Ref.		Ref.		23.2 (4.9)	Ref.		Ref.	
45-49	22.3 (4.8)	-1.2 (0.08)	<.0001	-1.3 (0.08)	<.0001	22.1 (4.7)	-1.1 (0.09)	<.0001	-1.1 (0.09)	<.0001
50-54	21.1 (4.7)	-2.4 (0.07)	<.0001	-2.4 (0.07)	<.0001	21.0 (4.6)	-2.2 (0.09)	<.0001	-2.2 (0.08)	<.0001
55-59	19.7 (4.8)	-3.8 (0.07)	<.0001	-3.7 (0.07)	<.0001	19.8 (4.5)	-3.4 (0.08)	<.0001	-3.4 (0.08)	<.0001
60-64	18.1 (4.8)	-5.4 (0.07)	<.0001	-5.1 (0.08)	<.0001	18.3 (4.5)	-4.9 (0.08)	<.0001	-4.7 (0.08)	<.0001
65+	15.9 (4.9)	-7.6 (0.08)	<.0001	-7.1 (0.09)	<.0001	16.3 (4.6)	-6.9 (0.08)	<.0001	-6.7 (0.09)	<.0001
Trend		-1.5 (0.01)	<.0001	-1.3 (0.02)	<.0001		-1.4 (0.01)	<.0001	-1.3 (0.02)	<.0001

*Significant sex×age interactions were observed for fluid intelligence, reaction time, pairs matching and symbol digit substitution (all P<0.0001).
†Model 1: no adjustments
‡Model 2: adjusted for smoking, Townsend deprivation index, education, income, alcohol intake, physical activity, ethnicity, and employment status.
§Negative beta-coefficients for FI and SDS correspond to lower performance compared to <45.
¶Positive beta-coefficients for Pairs, RT, Trail A and Trail B correspond to lower performance compared to <45.
