# Supplementary material for: Age and cognitive decline in the UK Biobank
Source: PLoS One. 2019 Mar 18;14(3):e0213948. doi: 10.1371/journal.pone.0213948 (PMC6422276; doi:10.1371/journal.pone.0213948)
Supplement: S6 Table — (PDF) [file pone.0213948.s007.pdf]

Table S6. Education-Stratified Cross-Sectional Associations Between Age and Cognitive Function Tests Measured At Baseline (2006-10)*

	Education ≤ level 4 (n≤186,740)					Education level 4+ (n≤281,794)				
	Mean (SD) Score	Model 1†		Model 2‡		Mean (SD) Score	Model 1†		Model 2‡	
		β (SE)	P	β (SE)	P		β (SE)	P	β (SE)	P
**§Fluid Intelligence**										
<45	5.28 (2.10)	Ref.		Ref.		6.34 (2.23)	Ref.		Ref.	
45-49	5.26 (2.05)	-0.02 (0.04)	0.53	-0.07 (0.04)	0.07	6.33 (2.20)	-0.01 (0.03)	0.71	-0.05 (0.03)	0.06
50-54	5.34 (2.09)	0.06 (0.04)	0.12	0.02 (0.04)	0.60	6.46 (2.15)	0.12 (0.03)	<.0001	0.06 (0.02)	0.01
55-59	5.44 (2.05)	0.16 (0.03)	<.0001	0.16 (0.03)	<.0001	6.61 (2.14)	0.27 (0.03)	<.0001	0.20 (0.02)	<.0001
60-64	5.35 (1.99)	0.07 (0.03)	0.03	0.09 (0.03)	0.01	6.53 (2.08)	0.17 (0.02)	<.0001	0.11 (0.03)	<.0001
65+	5.08 (1.89)	-0.20 (0.03)	<.0001	-0.13 (0.04)	0.00	6.19 (2.00)	-0.17 (0.03)	<.0001	-0.23 (0.03)	<.0001
Trend		-0.03 (0.01)	<.0001	0.0005 (0.01)	0.94		-0.005 (0.004)	0.27	0.002 (0.01)	0.79
**¶Pairs Matching**										
<45	1.34 (0.63)	Ref.		Ref.		1.28 (0.63)	Ref.		Ref.	
45-49	1.41 (0.62)	0.07 (0.007)	<.0001	0.07 (0.007)	<.0001	1.35 (0.63)	0.07 (0.005)	<.0001	0.07 (0.005)	<.0001
50-54	1.47 (0.62)	0.13 (0.006)	<.0001	0.13 (0.006)	<.0001	1.40 (0.63)	0.12 (0.004)	<.0001	0.12 (0.004)	<.0001
55-59	1.51 (0.61)	0.17 (0.006)	<.0001	0.17 (0.006)	<.0001	1.44 (0.62)	0.16 (0.004)	<.0001	0.17 (0.004)	<.0001
60-64	1.56 (0.61)	0.22 (0.006)	<.0001	0.22 (0.006)	<.0001	1.50 (0.62)	0.22 (0.004)	<.0001	0.23 (0.005)	<.0001
65+	1.65 (0.60)	0.31 (0.006)	<.0001	0.31 (0.007)	<.0001	1.58 (0.62)	0.31 (0.005)	<.0001	0.31 (0.006)	<.0001
Trend		0.06 (0.001)	<.0001	0.06 (0.001)	<.0001		0.06 (0.001)	<.0001	0.06 (0.001)	<.0001
**¶Reaction Time**										
<45	513.4 (100.4)	Ref.		Ref.		498.0 (86.6)	Ref.		Ref.	
45-49	531.7 (104.8)	17.9 (1.19)	<.0001	19.1 (1.18)	<.0001	514.6 (91.6)	16.2 (0.73)	<.0001	17.3 (0.72)	<.0001
50-54	547.6 (107.0)	33.7 (1.15)	<.0001	34.7 (1.14)	<.0001	533.3 (96.8)	34.9 (0.71)	<.0001	36.4 (0.70)	<.0001
55-59	565.5 (109.2)	51.3 (1.10)	<.0001	50.8 (1.10)	<.0001	550.1 (101.3)	52.1 (0.69)	<.0001	53.3 (0.70)	<.0001
60-64	585.7 (113.2)	71.5 (1.03)	<.0001	68.8 (1.12)	<.0001	569.6 (106.1)	72.4 (0.67)	<.0001	72.5 (0.74)	<.0001
65+	607.4 (117.3)	93.8 (1.04)	<.0001	87.9 (1.24)	<.0001	589.7 (112.7)	93.3 (0.73)	<.0001	92.3 (0.87)	<.0001
Trend		18.8 (0.16)	<.0001	17.2 (0.22)	<.0001		18.7 (0.12)	<.0001	18.3 (0.15)	<.0001
**¶Trail A**										
<45	3.49 (0.32)	Ref.		Ref.		3.43 (0.30)	Ref.		Ref.	
45-49	3.55 (0.32)	0.06 (0.009)	<.0001	0.07 (0.009)	<.0001	3.49 (0.30)	0.05 (0.004)	<.0001	0.06 (0.004)	<.0001
50-54	3.58 (0.33)	0.10 (0.008)	<.0001	0.10 (0.008)	<.0001	3.54 (0.30)	0.11 (0.004)	<.0001	0.11 (0.004)	<.0001
55-59	3.64 (0.32)	0.16 (0.008)	<.0001	0.16 (0.008)	<.0001	3.60 (0.31)	0.17 (0.004)	<.0001	0.17 (0.004)	<.0001

60-64	3.71 (0.32)	0.23 (0.008)	<.0001	0.21 (0.008)	<.0001	3.67 (0.30)	0.24 (0.004)	<.0001	0.23 (0.005)	<.0001
65+	3.81 (0.33)	0.33 (0.008)	<.0001	0.31 (0.010)	<.0001	3.75 (0.32)	0.33 (0.005)	<.0001	0.31 (0.005)	<.0001
Trend		0.06 (0.001)	<.0001	0.06 (0.002)	<.0001		0.07 (0.001)	<.0001	0.06 (0.001)	<.0001
**¶Trail B**										
<45	4.00 (0.31)	Ref.		Ref.		3.92 (0.30)	Ref.		Ref.	
45-49	4.06 (0.31)	0.06 (0.009)	<.0001	0.06 (0.009)	<.0001	3.98 (0.30)	0.06 (0.005)	<.0001	0.06 (0.004)	<.0001
50-54	4.12 (0.32)	0.12 (0.008)	<.0001	0.12 (0.008)	<.0001	4.04 (0.30)	0.12 (0.004)	<.0001	0.13 (0.004)	<.0001
55-59	4.20 (0.32)	0.20 (0.008)	<.0001	0.20 (0.008)	<.0001	4.12 (0.31)	0.20 (0.004)	<.0001	0.20 (0.004)	<.0001
60-64	4.30 (0.32)	0.30 (0.008)	<.0001	0.29 (0.008)	<.0001	4.21 (0.31)	0.29 (0.004)	<.0001	0.28 (0.005)	<.0001
65+	4.42 (0.34)	0.41 (0.008)	<.0001	0.40 (0.010)	<.0001	4.31 (0.32)	0.40 (0.005)	<.0001	0.38 (0.005)	<.0001
Trend		0.08 (0.001)	<.0001	0.08 (0.002)	<.0001		0.08 (0.001)	<.0001	0.07 (0.001)	<.0001
**§Symbol Digit Substitution**										
<45	22.6 (5.1)	Ref.		Ref.		23.6 (4.8)	Ref.		Ref.	
45-49	21.4 (4.9)	-1.2 (0.13)	<.0001	-1.2 (0.13)	<.0001	22.4 (4.6)	-1.2 (0.06)	<.0001	-1.2 (0.06)	<.0001
50-54	20.4 (5.0)	-2.3 (0.12)	<.0001	-2.3 (0.12)	<.0001	21.3 (4.5)	-2.2 (0.06)	<.0001	-2.3 (0.06)	<.0001
55-59	19.1 (5.0)	-3.5 (0.12)	<.0001	-3.5 (0.12)	<.0001	20.0 (4.6)	-3.6 (0.06)	<.0001	-3.6 (0.06)	<.0001
60-64	17.5 (4.9)	-5.1 (0.11)	<.0001	-5.0 (0.12)	<.0001	18.5 (4.5)	-5.1 (0.06)	<.0001	-4.9 (0.06)	<.0001
65+	15.4 (5.0)	-7.1 (0.12)	<.0001	-6.9 (0.14)	<.0001	16.5 (4.6)	-7.1 (0.07)	<.0001	-6.8 (0.08)	<.0001
Trend		-1.4 (0.02)	<.0001	-1.3 (0.02)	<.0001		-1.4 (0.01)	<.0001	-1.3 (0.01)	<.0001

*Significant education×age interactions were observed for fluid intelligence (P=0.005) and Trail B (P<0.0001)
†Model 1: adjusted for sex
‡Model 2: adjusted for sex, smoking, Townsend deprivation index, income, alcohol intake, physical activity, ethnicity, and employment status.
§Negative beta-coefficients for FI and SDS correspond to lower performance compared to <45.
¶Positive beta-coefficients for Pairs, RT, Trail A and Trail B correspond to lower performance compared to <45.
