# Supplementary material for: Age and cognitive decline in the UK Biobank
Source: PLoS One. 2019 Mar 18;14(3):e0213948. doi: 10.1371/journal.pone.0213948 (PMC6422276; doi:10.1371/journal.pone.0213948)
Supplement: S7 Table — (PDF) [file pone.0213948.s008.pdf]

Table S7. Apoe-Stratified Cross-Sectional Associations Between Age and Cognitive Function Tests Measured At Baseline (2006-10)*

	Apoe ε4 non-carriers (n≤273,846)					Apoe ε4 carriers (n≤108,567)				
	Mean (SD) Score	Model 1†		Model 2‡		Mean (SD) Score	Model 1†		Model 2‡	
		β (SE)	P	β (SE)	P		β (SE)	P	β (SE)	P
**§Fluid Intelligence**										
<45	5.98 (2.24)	Ref.		Ref.		6.02 (2.24)	Ref.		Ref.	
45-49	5.97 (2.22)	0.00 (0.03)	0.93	-0.03 (0.03)	0.310	6.04 (2.20)	0.02 (0.05)	0.600	-0.03 (0.04)	0.423
50-54	6.07 (2.21)	0.10 (0.03)	0.001	0.06 (0.03)	0.030	6.16 (2.19)	0.15 (0.04)	0.001	0.10 (0.04)	0.013
55-59	6.21 (2.20)	0.23 (0.03)	<.0001	0.22 (0.03)	<.0001	6.25 (2.17)	0.24 (0.04)	<.0001	0.22 (0.04)	<.0001
60-64	6.05 (2.13)	0.07 (0.03)	0.005	0.15 (0.03)	<.0001	6.04 (2.11)	0.02 (0.04)	0.626	0.08 (0.04)	0.053
65+	5.65 (2.02)	-0.34 (0.03)	<.0001	-0.16 (0.03)	<.0001	5.63 (2.03)	-0.41 (0.04)	<.0001	-0.22 (0.05)	<.0001
Trend		-0.05 (0.00)	<.0001	0.01 (0.01)	0.189		-0.07 (0.01)	<.0001	-0.01 (0.01)	0.551
**¶Pairs Matching**										
<45	1.30 (0.63)	Ref.		Ref.		1.30 (0.63)	Ref.		Ref.	
45-49	1.37 (0.63)	0.07 (0.005)	<.0001	0.07 (0.005)	<.0001	1.37 (0.63)	0.07 (0.008)	<.0001	0.07 (0.008)	<.0001
50-54	1.42 (0.63)	0.12 (0.005)	<.0001	0.13 (0.005)	<.0001	1.43 (0.63)	0.13 (0.008)	<.0001	0.13 (0.008)	<.0001
55-59	1.46 (0.62)	0.16 (0.005)	<.0001	0.16 (0.005)	<.0001	1.47 (0.62)	0.17 (0.007)	<.0001	0.17 (0.007)	<.0001
60-64	1.52 (0.62)	0.23 (0.004)	<.0001	0.22 (0.005)	<.0001	1.54 (0.61)	0.23 (0.007)	<.0001	0.23 (0.008)	<.0001
65+	1.62 (0.61)	0.32 (0.005)	<.0001	0.31 (0.006)	<.0001	1.62 (0.61)	0.32 (0.007)	<.0001	0.31 (0.009)	<.0001
Trend		0.06 (0.001)	<.0001	0.06 (0.001)	<.0001		0.06 (0.001)	<.0001	0.06 (0.002)	<.0001
**¶Reaction Time**										
<45	502.5 (91.1)	Ref.		Ref.		504.6 (92.5)	Ref.		Ref.	
45-49	519.3 (96.0)	16.5 (0.82)	<.0001	17.4 (0.81)	<.0001	522.0 (98.4)	17.0 (1.30)	<.0001	18.2 (1.28)	<.0001
50-54	538.1 (100.7)	34.9 (0.80)	<.0001	36.0 (0.79)	<.0001	537.8 (101.7)	32.6 (1.27)	<.0001	34.1 (1.26)	<.0001
55-59	554.6 (104.0)	51.6 (0.77)	<.0001	51.6 (0.78)	<.0001	557.0 (105.9)	51.9 (1.24)	<.0001	52.1 (1.24)	<.0001
60-64	576.7 (109.6)	74.0 (0.74)	<.0001	71.4 (0.81)	<.0001	575.0 (109.0)	70.2 (1.18)	<.0001	67.6 (1.30)	<.0001
65+	598.8 (115.9)	96.8 (0.77)	<.0001	91.2 (0.93)	<.0001	598.7 (115.2)	94.8 (1.23)	<.0001	89.0 (1.49)	<.0001
Trend		19.4 (0.12)	<.0001	18.0 (0.16)	<.0001		18.8 (0.20)	<.0001	17.4 (0.26)	<.0001
**¶Trail A**										
<45	3.44 (0.31)	Ref.		Ref.		3.44 (0.31)	Ref.		Ref.	
45-49	3.50 (0.31)	0.06 (0.005)	<.0001	0.06 (0.005)	<.0001	3.50 (0.30)	0.06 (0.008)	<.0001	0.06 (0.008)	<.0001
50-54	3.54 (0.31)	0.10 (0.005)	<.0001	0.10 (0.005)	<.0001	3.56 (0.31)	0.11 (0.008)	<.0001	0.11 (0.008)	<.0001
55-59	3.60 (0.31)	0.16 (0.005)	<.0001	0.16 (0.005)	<.0001	3.62 (0.31)	0.18 (0.008)	<.0001	0.17 (0.008)	<.0001

60-64	3.68 (0.31)	0.24 (0.005)	<.0001	0.22 (0.005)	<.0001	3.68 (0.31)	0.24 (0.008)	<.0001	0.22 (0.008)	<.0001
65+	3.77 (0.32)	0.33 (0.005)	<.0001	0.30 (0.006)	<.0001	3.78 (0.32)	0.34 (0.008)	<.0001	0.32 (0.01)	<.0001
Trend		0.07 (0.001)	<.0001	0.06 (0.001)	<.0001		0.07 (0.001)	<.0001	0.06 (0.002)	<.0001
**¶Trail B**										
<45	3.94 (0.31)	Ref.		Ref.		3.93 (0.30)	Ref.		Ref.	
45-49	4.00 (0.30)	0.06 (0.005)	<.0001	0.06 (0.005)	<.0001	4.00 (0.30)	0.06 (0.008)	<.0001	0.06 (0.008)	<.0001
50-54	4.05 (0.31)	0.11 (0.005)	<.0001	0.12 (0.005)	<.0001	4.07 (0.32)	0.14 (0.008)	<.0001	0.14 (0.008)	<.0001
55-59	4.13 (0.31)	0.19 (0.005)	<.0001	0.19 (0.005)	<.0001	4.15 (0.31)	0.22 (0.008)	<.0001	0.21 (0.008)	<.0001
60-64	4.23 (0.32)	0.29 (0.005)	<.0001	0.28 (0.005)	<.0001	4.24 (0.32)	0.31 (0.008)	<.0001	0.29 (0.008)	<.0001
65+	4.34 (0.33)	0.41 (0.005)	<.0001	0.38 (0.006)	<.0001	4.36 (0.34)	0.43 (0.009)	<.0001	0.41 (0.01)	<.0001
Trend		0.08 (0.001)	<.0001	0.07 (0.001)	<.0001		0.08 (0.001)	<.0001	0.08 (0.002)	<.0001
**§Symbol Digit Substitution**										
<45	23.4 (4.9)	Ref.		Ref.		23.4 (4.9)	Ref.		Ref.	
45-49	22.2 (4.7)	-1.2 (0.08)	<.0001	-1.2 (0.07)	<.0001	22.1 (4.7)	-1.3 (0.12)	<.0001	-1.4 (0.12)	<.0001
50-54	21.2 (4.7)	-2.2 (0.07)	<.0001	-2.2 (0.07)	<.0001	21.0 (4.7)	-2.5 (0.12)	<.0001	-2.5 (0.12)	<.0001
55-59	19.9 (4.7)	-3.5 (0.07)	<.0001	-3.4 (0.07)	<.0001	19.6 (4.7)	-3.8 (0.11)	<.0001	-3.7 (0.11)	<.0001
60-64	18.3 (4.6)	-5.1 (0.07)	<.0001	-4.8 (0.07)	<.0001	18.0 (4.7)	-5.4 (0.11)	<.0001	-5.2 (0.12)	<.0001
65+	16.3 (4.7)	-7.1 (0.07)	<.0001	-6.7 (0.09)	<.0001	15.7 (4.9)	-7.7 (0.12)	<.0001	-7.3 (0.14)	<.0001
Trend		-1.4 (0.01)	<.0001	-1.3 (0.01)	<.0001		-1.5 (0.02)	<.0001	-1.4 (0.02)	<.0001
**§Prospective Memory Test**										
	**% correct**	**OR (95% CI)**	**P**	**OR (95% CI)**	**P**	Mean	**OR (95% CI)**	**P**	**OR (95% CI)**	**P**
<45	80.8	Ref.		Ref.		80.9				
45-49	80.6	0.99 (0.93,1.06)	0.75	0.96 (0.90,1.03)	0.28	81.6	1.05 (0.94,1.16)	0.42	1.00 (0.90,1.11)	0.9785
50-54	79.2	0.91 (0.85,0.97)	0.003	0.86 (0.80,0.92)	<.0001	79.5	0.92 (0.83,1.01)	0.09	0.87 (0.78,0.97)	0.0091
55-59	78.5	0.87 (0.82,0.93)	<.0001	0.83 (0.78,0.88)	<.0001	79.5	0.92 (0.83,1.01)	0.08	0.86 (0.78,0.96)	0.0049
60-64	75.6	0.74 (0.70,0.78)	<.0001	0.71 (0.66,0.76)	<.0001	76.0	0.75 (0.68,0.82)	<.0001	0.72 (0.65,0.80)	<.0001
65+	69.1	0.53 (0.50,0.56)	<.0001	0.53 (0.49,0.57)	<.0001	68.4	0.51 (0.46,0.56)	<.0001	0.52 (0.46,0.58)	<.0001
Trend		0.88 (0.87,0.89)	<.0001	0.89 (0.88, 0.90)	<.0001		0.87 (0.86,0.88)	<.0001	0.89 (0.87,0.90)	<.0001

*Significant Apoe×age interactions were observed for fluid intelligence (P=0.02), reaction time (P=0.001), Trail B (P=0.01), and symbol digit substitution (P<0.0001).
†Model 1: adjusted for sex
‡Model 2: adjusted for sex, smoking, education, Townsend deprivation index, income, alcohol intake, physical activity, ethnicity, and employment status.
§Negative beta-coefficients for FI and SDS and OR <1 for PM correspond to lower performance compared to <45.
¶Positive beta-coefficients for Pairs, RT, Trail A and Trail B correspond to lower performance compared to <45.
