# Supplementary material for: Age and cognitive decline in the UK Biobank
Source: PLoS One. 2019 Mar 18;14(3):e0213948. doi: 10.1371/journal.pone.0213948 (PMC6422276; doi:10.1371/journal.pone.0213948)
Supplement: S8 Table — (PDF) [file pone.0213948.s009.pdf]

Table S8. Baseline Characteristics of Longitudinal Study Population*

Characteristic	Age Categories					
	**<45 (n=2254)**	**45-49 (n=3195)**	**50-54 (n=4077)**	**55-59 (n=5776)**	**60-64 (n=6895)**	**65+ (n=3808)**
Mean (SD) age, years	42.4 (1.3)	47.0 (1.4)	52.1 (1.4)	57.1 (1.4)	61.8 (1.4)	66.8 (1.5)
Female	1195 (53.0)	1767 (55.3)	2330 (57.2)	3115 (53.9)	3424 (49.7)	1645 (43.2)
White	2103 (93.3)	3034 (95.0)	3944 (96.7)	5660 (98.0)	6795 (98.6)	3753 (98.6)
Current smoking	226 (10.0)	288 (9.0)	272 (6.7)	375 (6.5)	365 (5.3)	177 (4.7)
Income						
<18,000	186 (8.3)	277 (8.7)	380 (9.3)	672 (11.6)	1264 (18.3)	918 (24.1)
18,000- 30,999	358 (15.9)	492 (15.4)	723 (17.7)	1212 (21.0)	2087 (30.3)	1299 (34.1)
31,000- 51,999	708 (31.4)	964 (30.2)	1180 (28.9)	1648 (28.5)	1715 (24.9)	741 (19.5)
52,000- 100,000	709 (31.5)	999 (31.3)	1251 (30.7)	1414 (24.5)	879 (12.8)	234 (6.1)
'100,000+	155 (6.9)	256 (8.0)	272 (6.7)	316 (5.5)	159 (2.3)	37 (1.0)
will not answer, missing	138 (6.1)	207 (6.5)	271 (6.7)	514 (8.9)	791 (11.5)	579 (15.2)
Education, level 4+	1729 (76.7)	2424 (75.9)	3100 (76.0)	4373 (75.7)	4756 (69.0)	2396 (63.0)
Employment status						
currently employed	2071 (91.9)	2916 (91.3)	3540 (86.8)	4129 (71.5)	2675 (38.8)	561 (14.7)
retired	3 (0.1)	21 (0.7)	195 (4.8)	1147 (19.9)	3987 (57.8)	3198 (84.0)
other/not reported	180 (8.0)	258 (8.1)	342 (8.4)	500 (8.7)	233 (3.4)	49 (1.3)
Mean (SD) Townsend deprivation score	-1.4 (3.0)	-1.6 (2.9)	-1.8 (2.8)	-2.1 (2.5)	-2.3 (2.5)	-2.3 (2.5)
Mean (SD) moderate to vigorous physical activity minutes/week	71.5 (83.6)	74.1 (99.1)	67.5 (80.6)	69.7 (80.6)	81.3 (90.3)	87.8 (99.0)
Mean (SD) alcohol drinks/day	1.1 (1.3)	1.2 (1.3)	1.2 (1.3)	1.3 (1.5)	1.2 (1.3)	1.2 (1.3)
Apoe ε4 carriers	507 (27.2)	779 (29.4)	910 (26.7)	1345 (28.2)	1592 (28.0)	869 (27.9)
Attended first follow-up (2012-13)	1438 (63.8)	2021 (63.26)	2709 (66.45)	4175 (72.28)	5262 (76.32)	2992 (78.57)
Attended second follow-up (2014+, 2015)	1177 (81.85)	1599 (79.12)	1941 (71.65)	2477 (59.33)	2509 (47.68)	1234 (41.24)

*Study population with baseline and up to 2 follow-up cognition assessments. Data drawn from 2006-10 (baseline). Values are numbers (percentages) unless stated otherwise. All characteristic values are significantly different across age-categories (P<0.0001) with the exception of Apoe (P=0.46).
