# Supplementary material for: Age and cognitive decline in the UK Biobank
Source: PLoS One. 2019 Mar 18;14(3):e0213948. doi: 10.1371/journal.pone.0213948 (PMC6422276; doi:10.1371/journal.pone.0213948)
Supplement: S9 Table — (PDF) [file pone.0213948.s010.pdf]

Table S9. Cross-sectional associations between age and cognitive function tests measured at baseline (2006-10) for follow-up sample and for full sample[a]

	Follow-up Sample		Full Sample (Table 2)	
	β (95% CI)	*P* value	β (95% CI)	*P* value
[c]Fluid Intelligence (n[b]=7563/158,673)				
<45	Reference		Reference	
45-49	-0.03 (-0.23,0.18)	0.81	-0.05 (-0.09,-0.01)	0.01
50-54	0.06 (-0.13,0.26)	0.53	0.05 (0.01,0.09)	0.01
55-59	0.18 (-0.01,0.37)	0.06	0.19 (0.16,0.23)	<.0001
60-64	0.15 (-0.05,0.35)	0.13	0.11 (0.07,0.15)	<.0001
65+	-0.23 (-0.46,-0.01)	0.04	-0.19 (-0.23,-0.14)	<.0001
Trend	-0.01 (-0.04,0.03)	0.79	0.002 (-0.01,0.01)	0.64
[de]Pairs Matching (n[b]=25,755/461,393)				
<45	Reference		Reference	
45-49	0.07 (0.04,0.11)	<.0001	0.07 (0.06,0.08)	<.0001
50-54	0.12 (0.09,0.15)	<.0001	0.13 (0.12,0.13)	<.0001
55-59	0.17 (0.14,0.20)	<.0001	0.17 (0.16,0.17)	<.0001
60-64	0.22 (0.19,0.26)	<.0001	0.22 (0.22,0.23)	<.0001
65+	0.30 (0.27,0.34)	<.0001	0.31 (0.30,0.32)	<.0001
Trend	0.06 (0.05,0.06)	<.0001	0.06 (0.06,0.06)	<.0001
[d]Reaction Time (n[b]=25,877/468,534)				
<45	Reference		Reference	
45-49	15.0 (9.8,20.2)	<.0001	17.9 (16.7,19.1)	<.0001
50-54	30.9 (25.9,35.8)	<.0001	35.9 (34.7,37.1)	<.0001
55-59	50.6 (45.9,55.4)	<.0001	52.4 (51.3,53.6)	<.0001
60-64	66.7 (61.7,71.7)	<.0001	71.0 (69.8,72.3)	<.0001
65+	83.9 (78.1,89.7)	<.0001	90.8 (89.4,92.2)	<.0001
Trend	17.0 (16.0,18.0)	<.0001	17.9 (17.7,18.2)	<.0001
[de]Trail A (n=12,596/100,354)				
<45	Reference		Reference	
45-49	0.07 (0.05,0.09)	<.0001	0.06 (0.05,0.07)	<.0001
50-54	0.11 (0.09,0.14)	<.0001	0.11 (0.10,0.11)	<.0001
55-59	0.18 (0.16,0.20)	<.0001	0.17 (0.16,0.17)	<.0001
60-64	0.24 (0.22,0.27)	<.0001	0.22 (0.22,0.23)	<.0001
65+	0.32 (0.30,0.35)	<.0001	0.31 (0.30,0.32)	<.0001
Trend	0.06 (0.06,0.07)	<.0001	0.06 (0.06,0.06)	<.0001
[de]Trail B (n[b]=12,696/100,352)				
<45	Reference		Reference	
45-49	0.06 (0.04,0.09)	<.0001	0.06 (0.05,0.07)	<.0001
50-54	0.13 (0.11,0.15)	<.0001	0.12 (0.12,0.13)	<.0001
55-59	0.20 (0.18,0.22)	<.0001	0.20 (0.19,0.20)	<.0001
60-64	0.28 (0.26,0.31)	<.0001	0.28 (0.27,0.29)	<.0001
65+	0.38 (0.36,0.41)	<.0001	0.39 (0.38,0.40)	<.0001
Trend	0.07 (0.07,0.08)	<.0001	0.07 (0.07,0.08)	<.0001

[c]Symbol Digit Substitution (n[b]=14,260/114,152)				
<45	Reference		Reference	
45-49	-0.8 (-1.1,-0.5)	<.0001	-1.2 (-1.3,-1.1)	<.0001
50-54	-2.1 (-2.4,-1.7)	<.0001	-2.3 (-2.4,-2.2)	<.0001
55-59	-3.2 (-3.5,-2.9)	<.0001	-3.6 (-3.7,-3.4)	<.0001
60-64	-4.8 (-5.1,-4.5)	<.0001	-4.9 (-5.1,-4.8)	<.0001
65+	-6.6 (-7.0,-6.2)	<.0001	-6.9 (-7.0,-6.8)	<.0001
Trend	-1.3 (-1.4,-1.2)	<.0001	-1.3 (-1.3,-1.3)	<.0001
[c]Prospective Memory Test (n[b]=7615/164,092)				
	OR (95% CI)	*P* value	OR (95% CI)	*P* value
<45	Reference		Reference	
45-49	0.91 (0.63,1.31)	0.59	0.96 (0.91, 1.01)	0.12
50-54	0.77 (0.55,1.08)	0.13	0.86 (0.81, 0.90)	<.0001
55-59	0.73 (0.52,1.01)	0.06	0.82 (0.78, 0.87)	<.0001
60-64	0.59 (0.42,0.82)	0.002	0.69 (0.66, 0.73)	<.0001
65+	0.47 (0.33,0.68)	<.0001	0.51 (0.49, 0.54)	<.0001
Trend	0.86 (0.81,0.92)	<.0001	0.89 (0.88, 0.89)	<.0001

[a]Shown are results from model 2 linear and logistic analysis (adjusted for sex, smoking, Townsend deprivation index, education, income, alcohol intake, physical activity, ethnicity, and employment status)
[b]Sample sizes for: follow-up sample / full sample
[c]Negative beta-coefficients for FI and SDS and OR <1 for PM correspond to lower performance compared to <45.
[d]Positive beta-coefficients for Pairs, RT, Trail A and Trail B correspond to lower performance compared to <45.
[e]Pairs Matching and Trail test scores were log transformed prior to analysis
