# Supplementary material for: Age and cognitive decline in the UK Biobank
Source: PLoS One. 2019 Mar 18;14(3):e0213948. doi: 10.1371/journal.pone.0213948 (PMC6422276; doi:10.1371/journal.pone.0213948)
Supplement: S10 Table — (PDF) [file pone.0213948.s011.pdf]

## Table S10. Sex-Stratified Longitudinal Analysis of Age and Cognitive Change

| | Women | | | | Men | | | |
|---|---|---|---|---|---|---|---|---|
| | Model 1* | | Model 2† | | Model 1* | | Model 2† | |
| | β (SE) | P | β (SE) | P | β (SE) | P | β (SE) | P |
| **§Fluid Intelligence** | | | | | | | | |
| <45 | Ref. | | Ref. | | Ref. | | Ref. | |
| 45-49 | 0.01 (0.02) | 0.55 | 0.01 (0.02) | 0.82 | 0.001 (0.03) | 0.98 | -0.001(0.03) | 0.96 |
| 50-54 | 0.01 (0.02) | 0.80 | -0.002 (0.02) | 0.94 | 0.03 (0.03) | 0.29 | 0.02 (0.03) | 0.43 |
| 55-59 | 0.01 (0.02) | 0.56 | 0.01 (0.02) | 0.80 | -0.02 (0.02) | 0.39 | -0.03 (0.02) | 0.29 |
| 60-64 | -0.02 (0.02) | 0.27 | -0.03 (0.02) | 0.31 | -0.03 (0.02) | 0.18 | -0.03 (0.02) | 0.27 |
| 65+ | -0.06 (0.03) | 0.02 | -0.06 (0.03) | 0.05 | -0.06 (0.03) | 0.03 | -0.04 (0.03) | 0.15 |
| *Trend* | -0.01 (0.004) | 0.002 | -0.01 (0.005) | 0.08 | -0.01 (0.004) | 0.0003 | -0.01 (0.005) | 0.04 |
| **¶Pairs Matching** | | | | | | | | |
| <45 | Ref. | | Ref. | | Ref. | | Ref. | |
| 45-49 | 0.01 (0.005) | 0.12 | 0.01 (0.005) | 0.09 | 0.01(0.01) | 0.33 | 0.01 (0.01) | 0.27 |
| 50-54 | 0.02 (0.004) | 0.00 | 0.02 (0.004) | <.0001 | 0.01(0.005) | 0.10 | 0.01 (0.005) | 0.06 |
| 55-59 | 0.02 (0.004) | <.0001 | 0.02 (0.004) | 0.0001 | 0.02(0.005) | 0.0004 | 0.02 (0.005) | 0.0002 |
| 60-64 | 0.03 (0.004) | <.0001 | 0.02 (0.005) | <.0001 | 0.02(0.005) | <.0001 | 0.02 (0.005) | <.0001 |
| 65+ | 0.04 (0.005) | <.0001 | 0.04 (0.006) | <.0001 | 0.04(0.005) | <.0001 | 0.04 (0.01) | <.0001 |
| *Trend* | 0.01 (0.001) | <.0001 | 0.01 (0.001) | <.0001 | 0.01(0.001) | <.0001 | 0.01 (0.001) | <.0001 |
| **¶Reaction Time** | | | | | | | | |
| <45 | Ref. | | Ref. | | Ref. | | Ref. | |
| 45-49 | 1.49 (0.62) | 0.02 | 1.59 (0.62) | 0.01 | 1.09 (0.67) | 0.10 | 1.12 (0.67) | 0.09 |
| 50-54 | 3.42 (0.59) | <.0001 | 3.45 (0.59) | <.0001 | 3.04 (0.64) | <.0001 | 3.17 (0.65) | <.0001 |
| 55-59 | 4.95 (0.57) | <.0001 | 4.80 (0.58) | <.0001 | 4.01 (0.61) | <.0001 | 4.15 (0.62) | <.0001 |
| 60-64 | 6.87 (0.58) | <.0001 | 6.58 (0.66) | <.0001 | 5.83 (0.59) | <.0001 | 5.88 (0.63) | <.0001 |
| 65+ | 8.03 (0.67) | <.0001 | 7.59 (0.79) | <.0001 | 7.29 (0.65) | <.0001 | 7.30 (0.74) | <.0001 |
| *Trend* | 1.67 (0.10) | <.0001 | 1.58 (0.14) | <.0001 | 1.48 (0.10) | <.0001 | 1.48 (0.13) | <.0001 |
| **§Prospective Memory Test** | | | | | | | | |
| | **OR (95% CI)** | **P** | **OR (95% CI)** | **P** | **OR (95% CI)** | **P** | **OR (95% CI)** | **P** |
| <45 | Ref. | | Ref. | | Ref. | | Ref. | |
| 45-49 | 1.09 (0.93,1.27) | 0.29 | 1.06 (0.90,1.25) | 0.48 | 0.90 (0.77,1.06) | 0.26 | 0.89 (0.75,1.06) | 0.19 |

| | | | | | | | | |
|---|---|---|---|---|---|---|---|---|
| 50-54 | 0.99 (0.86,1.13) | 0.84 | 0.96 (0.83,1.11) | 0.54 | 0.92 (0.78,1.08) | 0.40 | 0.89 (0.75,1.05) | 0.17 |
| 55-59 | 1.09 (0.95,1.26) | 0.24 | 1.06 (0.91,1.22) | 0.46 | 0.92 (0.79,1.05) | 0.27 | 0.87 (0.75,1.02) | 0.08 |
| 60-64 | 1.00 (0.87,1.15) | 0.96 | 0.98 (0.84,1.14) | 0.79 | 0.88 (0.77,1.01) | 0.06 | 0.87 (0.75,1.01) | 0.07 |
| 65+ | 0.97 (0.84,1.13) | 0.70 | 0.96 (0.79,1.15) | 0.64 | 0.83 (0.72, (0.95) | 0.01 | 0.80 (0.68, (0.95) | 0.01 |
| *Trend* | 0.99 (0.97,1.02) | 0.48 | 0.99 (0.96,1.03) | 0.67 | 0.97 (0.94,0.99) | 0.01 | 0.96 (0.94,0.99) | 0.06 |

Shown are results from linear mixed models with random intercept and time (slope):
*Model 1: included time, age, baseline test score, and all possible interactions with time. The time×age interaction term allows the calculation of the yearly rate of decline by age group with reference to the <45 age group.
†Model 2: included time, age, baseline test score, smoking, Townsend deprivation index, education, income, alcohol intake, physical activity, ethnicity, employment status, number of follow-up cognitive function tests completed, whether participants completed an on-line cognitive function test prior to the second follow-up (applicable to fluid intelligence and pairs matching tests only), and all possible interactions with time. The time×age interaction term allows the calculation of the yearly rate of decline by age group with reference to the <45 age group.
§Negative beta-coefficients for FI and OR <1 for PM correspond to declines in performance compared to <45.
¶Positive beta-coefficients for Pairs and RT correspond to declines in performance compared to <45.
