# Supplementary material for: Age and cognitive decline in the UK Biobank
Source: PLoS One. 2019 Mar 18;14(3):e0213948. doi: 10.1371/journal.pone.0213948 (PMC6422276; doi:10.1371/journal.pone.0213948)
Supplement: S11 Table — (PDF) [file pone.0213948.s012.pdf]

**Table S11. Education-Stratified Longitudinal Analysis of Age and Cognitive Change**

| | Education ≤ level 4 | | | | Education level 4+ | | | |
|---|---|---|---|---|---|---|---|---|
| | Model 1* | | Model 2† | | Model 1* | | Model 2† | |
| | β (SE) | P | β (SE) | P | β (SE) | P | β (SE) | P |
| **§Fluid Intelligence** | | | | | | | | |
| <45 | Ref. | | Ref. | | Ref. | | Ref. | |
| 45-49 | 0.00 (0.04) | 0.91 | -0.01 (0.04) | 0.79 | 0.01 (0.02) | 0.63 | 0.01 (0.02) | 0.80 |
| 50-54 | 0.03 (0.04) | 0.45 | 0.02 (0.04) | 0.55 | 0.01 (0.02) | 0.74 | 0.004 (0.02) | 0.84 |
| 55-59 | 0.02 (0.03) | 0.58 | 0.01 (0.03) | 0.67 | -0.01 (0.02) | 0.50 | -0.02 (0.02) | 0.37 |
| 60-64 | -0.02 (0.03) | 0.62 | -0.01 (0.03) | 0.75 | -0.03 (0.02) | 0.11 | -0.03 (0.02) | 0.15 |
| 65+ | -0.06 (0.03) | 0.08 | -0.05 (0.04) | 0.25 | -0.05 (0.02) | 0.03 | -0.05 (0.02) | 0.05 |
| *Trend* | -0.01 (0.01) | 0.02 | -0.01 (0.01) | 0.41 | -0.01 (0.003) | 0.0002 | -0.01 (0.004) | 0.01 |
| **‡¶Pairs Matching** | | | | | | | | |
| <45 | Ref. | | Ref. | | Ref. | | Ref. | |
| 45-49 | 0.01 (0.01) | 0.18 | 0.01 (0.01) | 0.13 | 0.01 (0.004) | 0.19 | 0.01 (0.004) | 0.15 |
| 50-54 | 0.02 (0.01) | 0.01 | 0.02 (0.01) | 0.01 | 0.01 (0.004) | 0.002 | 0.01 (0.004) | 0.001 |
| 55-59 | 0.02 (0.01) | 0.0003 | 0.02 (0.01) | 0.0003 | 0.02 (0.004) | <.0001 | 0.02 (0.004) | <.0001 |
| 60-64 | 0.03 (0.01) | <.0001 | 0.03 (0.01) | <.0001 | 0.02 (0.004) | <.0001 | 0.02 (0.004) | <.0001 |
| 65+ | 0.05 (0.01) | <.0001 | 0.05 (0.01) | <.0001 | 0.04 (0.004) | <.0001 | 0.04 (0.005) | <.0001 |
| *Trend* | 0.01 (0.001) | <.0001 | 0.01 (0.001) | <.0001 | 0.01 (0.001) | <.0001 | 0.01 (0.001) | <.0001 |
| **¶Reaction Time** | | | | | | | | |
| <45 | Ref. | | Ref. | | Ref. | | Ref. | |
| 45-49 | 0.92 (0.97) | 0.34 | 1.03 (0.97) | 0.29 | 1.41 (0.51) | 0.01 | 1.50 (0.51) | 0.003 |
| 50-54 | 2.82 (0.93) | 0.003 | 2.94 (0.93) | 0.002 | 3.36 (0.49) | <.0001 | 3.41 (0.49) | <.0001 |
| 55-59 | 3.45 (0.88) | <.0001 | 3.38 (0.90) | 0.0002 | 4.83 (0.47) | <.0001 | 4.81 (0.48) | <.0001 |
| 60-64 | 5.70 (0.85) | <.0001 | 5.50 (0.92) | <.0001 | 6.46 (0.47) | <.0001 | 6.44 (0.52) | <.0001 |
| 65+ | 6.85 (0.91) | <.0001 | 6.53 (1.06) | <.0001 | 7.82 (0.54) | <.0001 | 7.80 (0.62) | <.0001 |
| *Trend* | 1.44 (0.14) | <.0001 | 1.33 (0.18) | <.0001 | 1.60 (0.09) | <.0001 | 1.59 (0.11) | <.0001 |
| **§Prospective Memory Test** | | | | | | | | |
| | **OR (95% CI)** | **P** | **OR (95% CI)** | **P** | **OR (95% CI)** | **P** | **OR (95% CI)** | **P** |
| <45 | Ref. | | Ref. | | Ref. | | Ref. | |
| 45-49 | 0.93 (0.76,1.15) | 0.51 | 0.93 (0.74,1.17) | 0.54 | 1.06 ( (0.91,1.23) | 0.44 | 1.03 (0.88,1.19) | 0.74 |
| 50-54 | 0.84 (0.70,1.02) | 0.08 | 0.80 (0.65,0.99) | 0.04 | 1.00 ( 0.87,1.13) | 0.94 | 0.95 (0.83,1.09) | 0.47 |

| | | | | | | | | |
|---|---|---|---|---|---|---|---|---|
| 55-59 | 0.95 (0.78,1.15) | 0.57 | 0.93 (0.75,1.16) | 0.53 | 1.04 ( 0.92,1.18) | 0.56 | 0.99 (0.87,1.13) | 0.89 |
| 60-64 | 0.86 (0.72,1.03) | 0.11 | 0.79 (0.64,0.98) | 0.03 | 0.98 ( 0.87,1.11) | 0.73 | 0.94 (0.83,1.08) | 0.40 |
| 65+ | 0.86 (0.71,1.03) | 0.11 | 0.80 (0.64,1.01) | 0.06 | 0.92 ( 0.81,1.05) | 0.23 | 0.89 (0.76,1.03) | 0.13 |
| *Trend* | 0.98 (0.95,1.01) | 0.19 | 0.97 (0.93,1.01) | 0.11 | 0.98 ( 0.96,1.00) | 0.09 | 0.98 (0.95,1.01) | 0.13 |

Shown are results from linear mixed models with random intercept and time (slope):
*Model 1: included time, age, sex, baseline test score, and all possible interactions with time. The time×age interaction term allows the calculation of the yearly rate of decline by age group with reference to the <45 age group.
†Model 2: included time, age, sex, baseline test score, smoking, Townsend deprivation index, income, alcohol intake, physical activity, ethnicity, employment status, number of follow-up cognitive function tests completed, whether participants completed an on-line cognitive function test prior to the second follow-up (applicable to fluid intelligence and pairs matching tests only), and all possible interactions with time. The time×age interaction term allows the calculation of the yearly rate of decline by age group with reference to the <45 age group.
‡A significant education×time×age interaction were observed for pairs matching (P=0.02)
§Negative beta-coefficients for FI and OR <1 for PM correspond to declines in performance compared to <45.
¶Positive beta-coefficients for Pairs and RT correspond to declines in performance compared to <45.
