# Supplementary material for: Age and cognitive decline in the UK Biobank
Source: PLoS One. 2019 Mar 18;14(3):e0213948. doi: 10.1371/journal.pone.0213948 (PMC6422276; doi:10.1371/journal.pone.0213948)
Supplement: S12 Table — (PDF) [file pone.0213948.s013.pdf]

Table S12. Apoe-Stratified Longitudinal Analysis of Age and Cognitive Change

	Apoe ε4 non-carriers				Apoe ε4 carriers			
	Model 1*		Model 2†		Model 1*		Model 2†	
	β (SE)	P	β (SE)	P	β (SE)	P	β (SE)	P
**§Fluid Intelligence**								
<45	Ref.		Ref.		Ref.		Ref.	
45-49	0.003 (0.02)	0.89	-0.01 (0.02)	0.77	-0.03 (0.04)	0.41	-0.03 (0.04)	0.44
50-54	0.01 (0.02)	0.68	0.003 (0.02)	0.88	-0.02 (0.04)	0.57	-0.02 (0.04)	0.63
55-59	0.01 (0.02)	0.80	-0.002 (0.02)	0.91	-0.05 (0.04)	0.17	-0.05 (0.04)	0.19
60-64	-0.03 (0.02)	0.18	-0.02 (0.02)	0.31	-0.09 (0.04)	0.01	-0.09 (0.04)	0.03
65+	-0.04 (0.02)	0.12	-0.03 (0.03)	0.31	-0.12 (0.04)	0.01	-0.11 (0.05)	0.02
Trend	-0.01 (0.004)	0.007	-0.01 (0.004)	0.24	-0.02 (0.01)	0.0001	-0.02 (0.01)	0.009
**¶Pairs Matching**								
<45	Ref.		Ref.		Ref.		Ref.	
45-49	0.001 (0.004)	0.74	0.002 (0.004)	0.63	0.01 (0.01)	0.04	0.01 (0.01)	0.04
50-54	0.01 (0.004)	0.003	0.01 (0.004)	0.001	0.01 (0.01)	0.03	0.01 (0.01)	0.03
55-59	0.02 (0.004)	<.0001	0.02 (0.004)	<.0001	0.01 (0.01)	0.05	0.01 (0.01)	0.10
60-64	0.02 (0.004)	<.0001	0.02 (0.004)	<.0001	0.03 (0.01)	<.0001	0.02 (0.01)	0.0009
65+	0.04 (0.004)	<.0001	0.04 (0.01)	<.0001	0.04 (0.01)	<.0001	0.04 (0.01)	<.0001
Trend	0.01 (0.001)	<.0001	0.01 (0.001)	<.0001	0.01 (0.001)	<.0001	0.01 (0.001)	0.0004
**¶Reaction Time**								
<45	Ref.		Ref.		Ref.		Ref.	
45-49	1.51 (0.58)	0.009	1.63 (0.58)	0.005	1.74 (0.96)	0.07	1.80 (0.97)	0.06
50-54	3.27 (0.55)	<.0001	3.43 (0.56)	<.0001	3.01 (0.92)	0.001	2.99 (0.93)	0.001
55-59	4.48 (0.53)	<.0001	4.56 (0.54)	<.0001	4.40 (0.89)	<.0001	4.43 (0.91)	<.0001
60-64	6.09 (0.52)	<.0001	5.94 (0.57)	<.0001	6.70 (0.89)	<.0001	6.88 (0.98)	<.0001
65+	7.21 (0.59)	<.0001	6.85 (0.69)	<.0001	8.02 (1.00)	<.0001	8.35 (1.15)	<.0001
Trend	1.46 (0.09)	<.0001	1.41 (0.12)	<.0001	1.63 (0.16)	<.0001	1.62 (0.20)	<.0001
**§Prospective Memory Test**								
	**OR (95% CI)**	**P**	**OR (95% CI)**	**P**	**OR (95% CI)**	**P**	**OR (95% CI)**	**P**
<45	Ref.		Ref.		Ref.		Ref.	
45-49	1.00 (0.87,1.16)	0.95	n/a‡	n/a	0.99 (0.76,1.29)	0.97	n/a	n/a
50-54	0.94 (0.82,1.07)	0.35	n/a	n/a	0.97 (0.77,1.22)	0.79	n/a	n/a

55-59	1.03 (0.90,1.18)	0.67	n/a	n/a	0.94 (0.75,1.19)	0.62	n/a	n/a
60-64	0.97 (0.85,1.11)	0.70	n/a	n/a	0.90 (0.73,1.12)	0.35	n/a	n/a
65+	0.93 (0.81,1.06)	0.27	n/a	n/a	0.80 (0.63,1.01)	0.06	n/a	n/a
Trend	0.99 (0.97,1.01)	0.36	0.99 (0.97,1.02)	0.70	0.96 (0.92,0.99)	0.03	0.96 (0.91,1.00)	0.05

Shown are results from linear mixed models with random intercept and time (slope):
*Model 1: included time, age, sex, baseline test score, and all possible interactions with time. The time×age interaction term allows the calculation of the yearly rate of decline by age group with reference to the <45 age group.
†Model 2: included time, age, sex, baseline test score, smoking, Townsend deprivation index, education, income, alcohol intake, physical activity, ethnicity, employment status, number of follow-up cognitive function tests completed, whether participants completed an on-line cognitive function test prior to the second follow-up (applicable to fluid intelligence and pairs matching tests only), and all possible interactions with time. The time×age interaction term allows the calculation of the yearly rate of decline by age group with reference to the <45 age group.
‡ n/a: statistical models did not converge.
§Negative beta-coefficients for FI correspond to declines in performance compared to <45.
¶Positive beta-coefficients for Pairs and RT correspond to declines in performance compared to <45.
