# Supplementary material for: Age and cognitive decline in the UK Biobank
Source: PLoS One. 2019 Mar 18;14(3):e0213948. doi: 10.1371/journal.pone.0213948 (PMC6422276; doi:10.1371/journal.pone.0213948)
Supplement: S13 Table — (PDF) [file pone.0213948.s014.pdf]

Table S13. Baseline-Cognition -Stratified Longitudinal Analysis of Age and Cognitive Change

	Low baseline (< median test score)				High baseline (≥ median test score)			
	Model 1*		Model 2†		Model 1*		Model 2†	
	β (SE)	P	β (SE)	P	β (SE)	P	β (SE)	P
**‡§Fluid Intelligence**								
<45	Ref.		Ref.		Ref.		Ref.	
45-49	0.02 (0.03)	0.47	0.02 (0.03)	0.56	-0.0001 (0.02)	1.00	-0.01 (0.02)	0.77
50-54	0.04 (0.03)	0.10	0.03 (0.03)	0.23	-0.01 (0.02)	0.66	-0.01 (0.02)	0.63
55-59	0.05 (0.03)	0.05	0.03 (0.03)	0.27	-0.05 (0.02)	0.04	-0.04 (0.02)	0.07
60-64	0.05 (0.03)	0.07	0.04 (0.03)	0.19	-0.08 (0.02)	0.0002	-0.07 (0.02)	0.005
65+	0.0001 (0.03)	0.99	-0.01 (0.03)	0.76	-0.10 (0.02)	<.0001	-0.08 (0.03)	0.003
Trend	0.001 (0.004)	0.87	0.001 (0.01)	0.84	-0.02 (0.004)	<.0001	-0.02 (0.005)	<.0001
**‡¶Pairs Matching**								
<45	Ref.		Ref.		Ref.		Ref.	
45-49	0.01 (0.004)	0.08	0.01 (0.004)	0.05	0.003 (0.01)	0.59	0.004 (0.01)	0.49
50-54	0.02 (0.004)	0.0004	0.02 (0.004)	0.0002	0.01 (0.01)	0.09	0.01 (0.01)	0.07
55-59	0.02 (0.004)	<.0001	0.02 (0.004)	<.0001	0.01 (0.005)	0.06	0.01 (0.005)	0.03
60-64	0.04 (0.004)	<.0001	0.03 (0.005)	<.0001	0.01 (0.005)	0.01	0.01 (0.01)	0.01
65+	0.05 (0.005)	<.0001	0.04 (0.006)	<.0001	0.03 (0.01)	<.0001	0.03 (0.01)	<.0001
Trend	0.01 (0.001)	<.0001	0.01 (0.001)	<.0001	0.01 (0.001)	<.0001	0.005 (0.001)	<.0001
**‡¶Reaction Time**								
<45	Ref.		Ref.		Ref.		Ref.	
45-49	1.31 (0.47)	0.01	1.38 (0.47)	0.003	0.52 (0.94)	0.58	0.54 (0.94)	0.57
50-54	3.10 (0.46)	<.0001	3.17 (0.46)	<.0001	2.72 (0.87)	0.002	2.75 (0.88)	0.002
55-59	4.25 (0.44)	<.0001	4.24 (0.45)	<.0001	3.92 (0.82)	<.0001	3.91 (0.83)	<.0001
60-64	6.53 (0.45)	<.0001	6.33 (0.50)	<.0001	5.62 (0.81)	<.0001	5.52 (0.86)	<.0001
65+	8.64 (0.55)	<.0001	8.25 (0.64)	<.0001	6.75 (0.85)	<.0001	6.59 (0.95)	<.0001
Trend	1.69 (0.08)	<.0001	1.58 (0.10)	<.0001	1.46 (0.12)	<.0001	1.42 (0.16)	<.0001

Shown are results from linear mixed models with random intercept and time (slope):
*Model 1: included time, age, sex, baseline test score, and all possible interactions with time. The time×age interaction term allows the calculation of the yearly rate of decline by age group with reference to the <45 age group.
†Model 2: included time, age, sex, baseline test score, smoking, Townsend deprivation index, education, income, alcohol intake, physical activity, ethnicity, employment status, number of follow-up cognitive function tests completed, whether participants completed an on-line cognitive function test prior to the second follow-up (applicable to fluid intelligence and pairs matching tests only), and all possible interactions with time. The time×age interaction term allows the calculation of the yearly rate of decline by age group with reference to the <45 age group.

‡Significant baseline score×time×age interaction were observed for fluid intelligence (P=0.03), pairs matching (P<0.0001) and reaction time (P=0.002)
§Negative beta-coefficients for FI correspond to declines in performance compared to <45.
¶Positive beta-coefficients for Pairs and RT correspond to declines in performance compared to <45.
