# Supplementary material for: Age and cognitive decline in the UK Biobank
Source: PLoS One. 2019 Mar 18;14(3):e0213948. doi: 10.1371/journal.pone.0213948 (PMC6422276; doi:10.1371/journal.pone.0213948)
Supplement: S14 Table — (PDF) [file pone.0213948.s015.pdf]

Table S14. Baseline Characteristics of 'Follow-Up Only' Study Population*

Characteristic	Age categories					
	**<45 (n=1234)**	**45-49 (n=1658)**	**50-54 (n=2171)**	**55-59 (n=3310)**	**60-64 (n=4022)**	**65+ (n=2235)**
Mean (SD) age, years	42.30 (1.3)	47.07 (1.4)	52.15 (1.4)	57.12 (1.4)	61.81 (1.4)	66.82 (1.5)
Female	650 (52.7)	906 (54.6)	1242 (57.2)	1785 (54.0)	2021 (50.3)	980 (43.9)
White	1153 (93.4)	1579 (95.2)	2089 (96.2)	3240 (97.9)	3961 (98.5)	2201 (98.5)
Current smoking	127 (10.3)	153 (9.2)	158 (7.3)	237 (7.2)	233 (5.8)	104 (4.7)
Income						
<18,000	101 (8.2)	136 (8.2)	218 (10.0)	395 (11.9)	769 (19.1)	591 (26.4)
18,000- 30,999	190 (15.4)	275 (16.6)	392 (18.1)	700 (21.2)	1211 (30.1)	725 (32.4)
31,000- 51,999	341 (27.6)	476 (28.7)	583 (26.9)	900 (27.2)	932 (23.1)	390 (17.5)
52,000- 100,000	378 (30.6)	479 (28.9)	622 (28.7)	747 (22.6)	475 (11.8)	105 (4.7)
'100,000+	86 (7.0)	140 (8.4)	126 (5.8)	179 (5.4)	82 (2.0)	15 (0.7)
will not answer, missing	138 (11.2)	152 (9.2)	230 (10.6)	389 (11.8)	556 (13.8)	409 (18.3)
Education, level 4+	926 (75.0)	1245 (75.1)	1575 (72.6)	2414 (72.9)	2639 (65.6)	1325 (59.3)
Employment status						
currently employed	1131 (91.7)	1523 (91.9)	1886 (86.9)	2377 (71.8)	1580 (39.3)	299 (13.4)
retired	2 (0.2)	10 (0.6)	101 (4.7)	646 (19.5)	2300 (57.2)	1904 (85.3)
other/not reported						
Mean (SD) Townsend deprivation score	-1.27 (3.0)	-1.47 (3.0)	-1.67 (2.9)	-2.02 (2.6)	-2.25 (2.6)	-2.22 (2.6)
Mean (SD) moderate to vigorous physical activity minutes/week	64.9 (78.2)	71.5 (110.0)	62.8 (81.4)	67.1 (81.6)	77.2 (90.3)	83.6 (97.9)
Mean (SD) alcohol drinks/**day**	1.13 (1.41)	1.18 (1.31)	1.20 (1.26)	1.26 (1.50)	1.25 (1.33)	1.21 (1.34)
Apoe ε4 carriers	267 (25.9)	400 (28.9)	541 (29.7)	781 (28.3)	944 (28.3)	523 (28.5)
FU 1 (2012-13, 2013), years	4.84 (0.67)	4.73 (0.65)	4.75 (0.67)	4.71 (0.64)	4.67 (0.62)	4.72 (0.63)

*Study population not completing baseline but the first follow-up cognition assessment. Data drawn from 2006-10 (baseline). Values are numbers (percentages) unless stated otherwise. All characteristic values are significantly different across age-categories (P<0.02) with the exception of Apoe (P=0.40).
